# Precise and Omnidirectional Opto‐Thermo‐Elastic Actuation in Van Der Waals Contacting Systems

**DOI:** 10.1002/advs.202401418

**Published:** 2024-08-19

**Authors:** Qiannan Jia, Renjie Tang, Xiaoyu Sun, Weiwei Tang, Lan Li, Jiajie Zhu, Pan Wang, Wei Yan, Min Qiu

**Affiliations:** ^1^ College of Information Science and Electronic Engineering Zhejiang University Hangzhou Zhejiang Province 310027 China; ^2^ Key Laboratory of 3D Micro/Nano Fabrication and Characterization of Zhejiang Province School of Engineering Westlake University Hangzhou Zhejiang Province 310024 China; ^3^ Institute of Advanced Technology Westlake Institute for Advanced Study Hangzhou Zhejiang Province 310024 China; ^4^ College of Physics and Optoelectronic Engineering Hangzhou Institute for Advanced Study University of Chinese Academy of Sciences Hangzhou Zhejiang Province 310024 China; ^5^ Westlake Institute for Optoelectronics Hangzhou Zhejiang Province 311421 China; ^6^ State Key Laboratory of Extreme Photonics and Instrumentation, College of Optical Science and Engineering Zhejiang University Hangzhou 310027 China

**Keywords:** elastic wave, friction force, optical manipulation, van der Waals contacts

## Abstract

Actuation of micro‐objects along unconstrained trajectories in van der Waals contacting systems—in the same capacity as optical tweezers to manipulate particles in fluidic environments—remains a formidable challenge due to the lack of effective methods to overcome and exploit surface friction. Herein, a technique that aims to resolve this difficulty is proposed. This study shows that, by utilizing a moderate power beam of light, micro‐objects adhered on planar solid substrates can be precisely guided to move in arbitrary directions, realizing sub‐nanometer resolution across extended surfaces. The underlying mechanism is the interplay between surface friction and pulsed opto‐thermo‐elastic deformations, and to render a biased motion with off‐centroid light illumination. This technique enables high‐precision assembly, separation control of nanogaps, regulation of rotation angles in various material‐substrate systems, whose capability is further tested in reconfigurable construction of optoelectronic devices. With simple set‐up and theoretical generality, opto‐thermo‐elastic actuation opens up an avenue for versatile optical manipulation in the solid domain.

## Graphical abstract

The article discusses the development of an all‐optical method to arbitrarily manipulate micron‐sized objects forming van der Waals contacts with the planar solid substrates. The proposed technique utilizes an opto‐thermo‐elastic coupled system, which can achieve sub‐nanometer spatial resolution and guide actuators to move in all directions on the contacting surface.

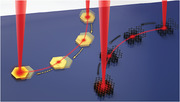

## 1 Introduction

Optical tweezer has been an essential tool for manipulating motion of tiny objects in fluidic environments, i.e., liquid,^[^ ^1^ ^]^ air,^[^ ^2^, ^3^ ^]^ and vacuum,^[^ ^4^, ^5^ ^]^ ever since its invention by Arthur Ashkin and his colleagues.^[^ ^6^, ^7^ ^]^ The widespread use of optical tweezer is rooted in its unique traits to operate without imposing strict requirements on either target particles or surrounding fluids, and at the same time achieve versatile manipulation with high precision. Extension of optical tweezers to planar solid surfaces, while preserving their competitive traits, is highly desirable for various science and technological scenarios that require solid platforms, such as optoelectronics,^[^ ^8^, ^9^, ^10^ ^]^ van der Waals devices,^[^ ^11^, ^12^ ^]^ and interface physics.^[^ ^13^, ^14^ ^]^ However, its implementation is hindered by the huge adhesive pressure of van der Waals contacts between solid surfaces (≈ 10^6^–10^8^ N m^−2^),^[^ ^15^, ^16^, ^17^, ^18^ ^]^ which overshadows the optical radiation pressure by roughly six orders of magnitude.^[^ ^6^ ^]^ The component of the van der Waals pressure perpendicular to the contacting interface contributes to adhesive force, which firmly anchors the micro‐objects and prevents their detachment. The component parallel to the contacting interface serves as the friction force, which inhibits the objects from moving along the contacting surface.

Rather than solely focusing on photon momentum with minute magnitude, exploitation of light energy to control multi‐physics induced mechanical effects brings inspiring opportunities for optical manipulation on solid interfaces.^[^ ^19^ ^]^ For instance, by photothermally inducing solid‐to‐liquid phase transition of substrates, surface friction can be minimized, thereby allowing optical force or capillary force‐driven actuation.^[^ ^20^, ^21^, ^22^ ^]^ Studies of soft robotics have realized multimode locomotion in various working environments in response to irradiation‐imposed deformations, where machinery parts are designed and assembled on actuators to imitate the function of macroscopic motors.^[^ ^23^, ^24^, ^25^, ^26^, ^27^, ^28^ ^]^ A few studies have reported the employment of pulsed laser‐excited elastic waves, which we first developed and tested on sidewall of micro‐fibers^[^ ^29^, ^30^, ^31^, ^32^ ^]^ and herein generalize as the opto‐thermo‐elastic (OTE) mechanism, in enabling locomotion of micro‐objects on solid surfaces. A recent study has demonstrated optical driving on planar solid substrates, which focuses exclusively on two‐dimensional materials. In this study, the actuation direction is pre‐defined by the asymmetry in the interfacial contacts, and hence is not subject to on‐demand adjustments.^[^ ^33^ ^]^ A more recent research has demonstrated the use of thermal gradient force to power solid domain metallic nanorobots.^[^ ^34^ ^]^ An irresistible trend has emerged: extending the optical manipulation capability beyond the fluidic domain and into systems with adhesive van der Waals contacts. Specifically, a universal technique in parallel to the optical tweezers, allowing for high‐precision, omnidirectional, versatile, and contact‐free manipulation of various micro‐materials in the solid domain, and is underpinned by clear physical pictures, is in urgent demand.

**Figure 1:**
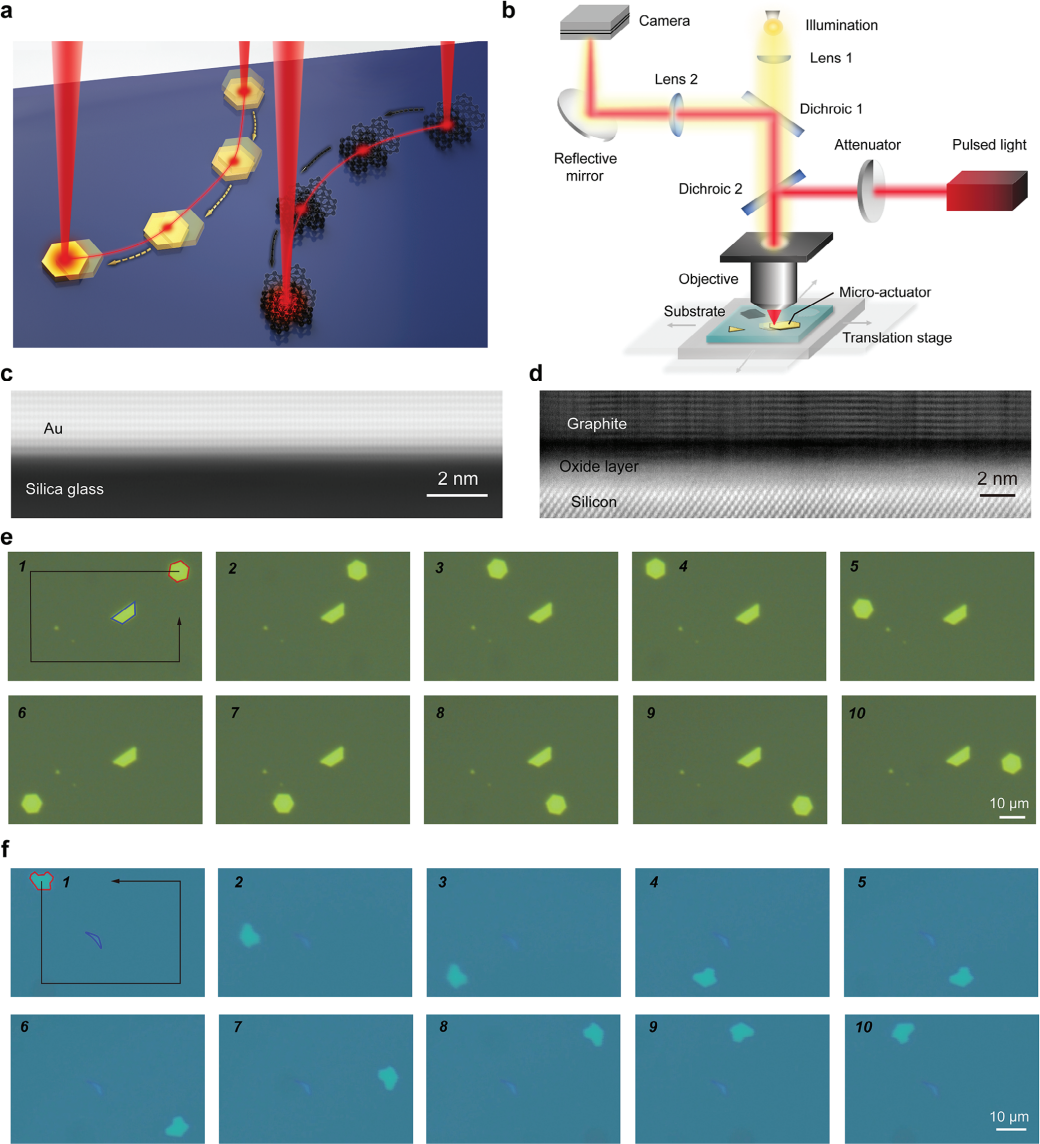
Omnidirectional opto‐thermo‐elastic actuation on solid substrates. a) Schematic of the directed actuation of gold plates and layered materials following predefined trajectories. Directionality of the actuation is derived from off‐centroid illumination by focused laser beams (wavelength, 1030 nm; pulse duration: 2 ns). b) Experimental set‐up of the proposed actuation scheme. HAADF‐STEM images showing cross‐sections of c) the gold plate–quartz glass interface and d) the graphite–silicon interface. Sequential microscopic images showing the directed motion of e) a target gold plate and f) a fragment of exfoliated pyrolytic graphite. In the first snapshots, the target objects are outlined in red, while the objects for reference are outlined in blue, and the arrows indicate the moving paths of the two materials. The average power and repetition rate of the light source are 125 µW and 1 kHz, respectively, for the gold plate, and 2.9 µW and 100 Hz, respectively, for the graphite flake. Utilizing the 20× objective, the focused Gaussian light spot has a 1/*e* diameter of 4.33 µm.

In pursuit of providing a powerful tool to address this demand, we here demonstrate precise and omnidirectional manipulation of micro‐objects on planar solid surfaces (**Figure** **1**a) by exploring the full potential of OTE actuation. The proposed technique only requires light absorption of target objects. To demonstrate its potential to be generally applied to different systems, we exemplify two sets of high‐modulus materials: gold micro‐plates and layered materials—including pyrolytic graphite and molybdenum disulfide (MoS_2_)—forming van der Waals contacts with various underlying substrates. Despite drastic distinctions among these systems, criteria of high spatial resolution, high‐speed locomotion and omnidirectional motion can be simultaneously fulfilled. A shared locomotion pattern and actuation mechanism exists, unveiling the key role of off‐centroid illumination. Theoretical insights are provided, elucidating the interaction between friction force and asymmetrically excited elastic waves at planar van der Waals contacts in enabling the directed OTE actuation. Utilizing the proposed technique, a top‐down route is unlocked for precise and rewritable van der Waals integration into functional optoelectronic devices, similarly to building blocks.

## 2 Results

### 2.1 Actuation Characteristics

Experimental configuration of the proposed OTE actuation technique is shown in Figure 1b. Through a vertically aligned optical path, a beam of nanosecond pulsed laser is focused into a diffraction‐limited Gaussian spot (see Figure S1, Supporting Information for light spot characterization) before it irradiates the target actuator. Upon irradiation, light‐absorptive materials produce transient photothermal effects and undergo intense thermal deformations due to excited elastic waves, which exhibit distinct characteristics when compared to static states under continuous wave (CW) light irradiation.^[^ ^35^, ^36^ ^]^ A pivotal factor here is to deviate the center of the light spot from the center of the target actuator, which implements the directional bias by virtue of the asymmetrically excited elastic waves. The van der Waals interaction between the actuator and the contacting substrate contributes to surface adhesion and friction that anchor the actuator at its initial position. The in‐plane component of the van der Waals force, i.e., the friction force, functions to resist the moving tendency of the actuator and reaches the scale of micronewtons when exerted on the micro‐sized actuators. The actuator can only start locomotion when the input optical power surpasses a certain threshold necessary to overcome the static friction force,^[^ ^30^ ^]^ the irradiated material would locomote in a direction that tends to reduce the bias, which always points from its own center of mass (centroid) toward the center of the light spot. The directional bias, i.e., the deviation between the light spot center and the actuator's centroid, can be adjusted and constantly restored by moving the translation stage that holds the planar substrate, equivalent to moving the light spot while keeping the sample position fixed. As a result, actuators can be continuously “dragged” along the trajectory of the light spot (utilizing the translation stage as the reference frame) till the directional bias ceases to be reintroduced, as schematically illustrated in Figure 1a. The actuation process can be observed in real time and recorded by the microscope camera.

To implement the OTE actuation, planar van der Waals contacts should be formed between target actuators and the underlying substrates. This ensures that the friction force can be generated omnidirectionally during locomotion. The contact configurations between actuators and planar substrates are unveiled by the high‐angle annular dark field (HAADF) scanning transmission electron microscopic (STEM) images in Figure 1c,d (more morphological information is provided in Figure S2, Supporting Information), which exhibit clear and atomically smooth interfaces. Figure 1e,f are sequential optical microscopic images that display the directed motion of a gold plate on a quartz glass and a pyrolytic graphite flake on a silicon substrate, respectively. The experimental parameters employed in the OTE actuation are detailed in the figure caption (as is the practice for all experimental results presented). Regardless of the distinctions such as the strength of the van der Waals contacts, light absorptivity, thermal conductivity, structural organization, etc., both materials (outlined in red) can be navigated to move along pre‐defined paths around the static reference materials (outlined in blue) with high fidelity by continuously updating the directional bias, thereby showcasing that the motion direction can be arbitrarily adjusted in real time (Movies S1 and S2, Supporting Information). Owing to the use of a moderate laser power, scanning electron microscopic (SEM) images and Raman spectroscopies suggest that the laser irradiation during the OTE manipulation does not induce either ablation or chemical degradation of materials (Figure S2, Supporting Information). Initiation of the observed translational motion (as well as the orientation change of materials, which corresponds to in‐plane rotations that will be discussed in Figure 4) requires the use of pulsed form lasers; in comparison, actuators remain strictly static under irradiation of CW light till ablated.

**Figure 2:**
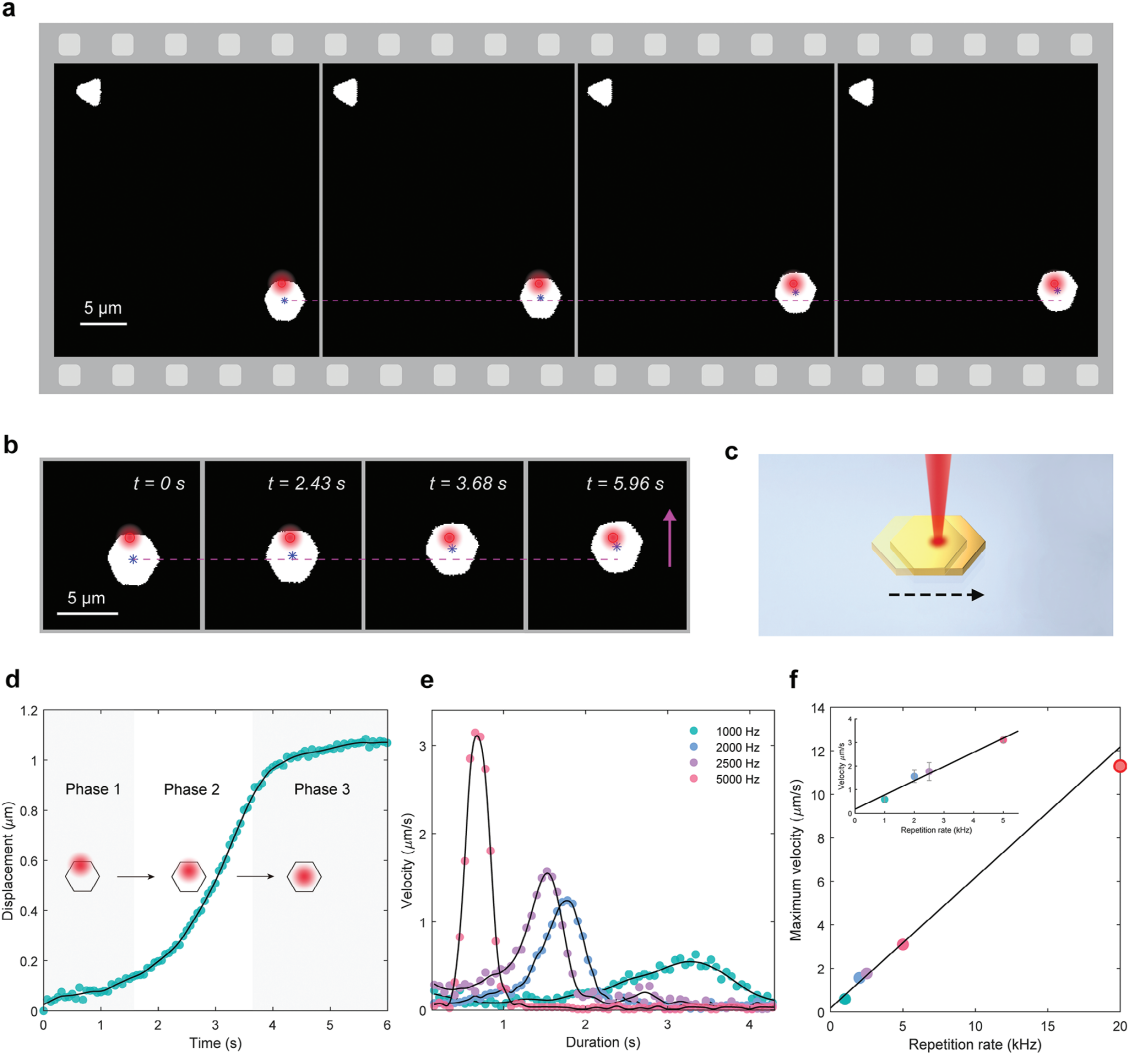
Dynamics of the OTE actuation. a) Successive optical images showing the centroid motion of the target gold plate (hexagonal) relative to the light spot. A reference plate (triangle) sitting at the upper left corner of each image is purposedly included in the frame to assist the alignment of different snapshots. The light spot center is highlighted in red, and the centroid of the actuator is marked with a blue asterisk. A magenta dashed line is drawn horizontally to facilitate the observation of the motor's movement. In this experiments, the 1/*e* diameter of the focused Gaussian light spot is 2.03 µm with the use of the 50X objective. b) Zoomed‐in snapshots in (a). c) Schematic illustrating the directionality of the OTE actuation. With appropriate deviation between the light spot center and the actuator's centroid, the motor would always locomote in a direction that inclines to reduce the directional bias. d) Displacement of the target gold plate as a function of time. The employed laser power and repetition rate are 81.2 µW and 1 kHz, respectively. Insets indicate the position evolution of the irradiated area on the gold plate at varying stages of the induced locomotion step. e) Velocity as a function of time of the target gold plate with different repetition rates of the driving light source. The single pulse energy is kept constant at 81.2 nJ. f) Relationship between the moving speed and repetition rate. The datapoint at 20 kHz is obtained by finding the lowest repetition rate that produces synchronization between the motion of the gold plate and the translation stage, the latter moving at a speed of 11 µm s^−1^. Inset shows the zoomed‐in linear fit of the four datapoints acquired using the centroid motion extraction analysis. Error bars suggest the level of variance among 5 measurements.

To unveil the dynamics of the light‐directed OTE actuation, videos are recorded during individual motion steps, which, for better visualization and clarity, are binarized and analyzed frame by frame, as shown in **Figure** **2**a. Note that in this context, the motion step refers to the duration between the inception and completion of the actuator's motion, preceded by a single adjustment of the directional bias. Consecutive images show that, with initial irradiation asymmetry, a hexagonal‐shaped gold plate would locomote to mitigate this asymmetry by transitioning toward the light spot (highlighted in red), and it ceases to move when its centroid (blue asterisk) is at close proximity to the light spot center (Figure 2b). Same results are reliably replicated in other moving directions and on other gold plate samples (see Figure S3, Supporting Information), suggesting a general pattern of OTE actuation guided by the directional bias, as depicted in Figure 2c. The systematic recurrence of the minor misalignment between the spot center and the actuator's centroid might be the result of chromatic aberration between the driving laser (1030 nm) and the illumination light source (broadband white light). For future reference, it can be minimized through post‐processing techniques to correct aberration or by utilizing achromatic lenses.

Corresponding to the process shown in Figure 2a,b, displacement of the gold plate's centroid over time is depicted in Figure 2d. The displacement versus time curve being nonlinear suggests nonuniform traveling speed. It features three distinct stages: the low‐speed initial phase, the high‐speed acceleration phase, and the concluding phase where the motion of the plate gradually comes to a halt. Insets in Figure 2d (the result of cross‐referencing Figure 2b,d) demonstrates the position evolution of the irradiated area on the gold plate throughout the three phases. Evidently, a large directional bias does not necessarily come with a high traveling speed. For high‐speed actuation, the optimal configuration is for the center of the Gaussian light spot to locate close to the midpoint between the edge and the centroid of the plate, with a preference for being closer to the former (explanations are provided later). Accordingly, if the gold plate commences its motion from the optimum configuration, there would be no acceleration or the low‐speed initial phase. Instead, the motor would start at a maximum speed and continuously decelerates till the motion ceases (Figure S4, Supporting Information).

The OTE actuation is driven by pulsed lasers. The number of pulses transmitted within a certain time interval, i.e., the repetition rate of laser pulses influences the velocity of motors, based on the premise that the neighboring pulses are mutually independent and that the locomotion is pulse wise.^[^ ^29^, ^30^ ^]^ Figure 2e shows the velocity evolution of the same gold plate probed at different repetition frequencies with similar initial settings (the relative position between the motor's centroid and the light spot, single pulse energy, etc.). A monotonically increasing relation is revealed between the traveling speed and the repetition rate at corresponding phases of acceleration and deceleration. Note that at a fixed repetition rate, the change of velocity signifies variation of the pulse‐wise step size among different light pulses. In this context, the pulse‐wise motion step represents the motion upon one single shot of light pulse, not to be confused with the previously discussed three‐phase motion step, the latter being composed of a train of light pulses. Hence, a more accurate interpretation of the velocity evolution is that the step size of the gold plate alters with each renewed level of absorption asymmetry during its movement. The step size per laser pulse at different time can be calculated as *d*_single_ = *v*(*t*)/*f* , where *v*(*t*) is the travelling velocity obtained by the recording device, and *f* is the repetition rate. The time‐averaged step size of the centroid's motion, representing the spatial resolution of the directed OTE actuation, can be estimated as ${\bar{d}}_{\mathrm{single}}=\;\Delta d/\left(\Delta t*f\right)$, where Δ*d* is the traveling distance during the time duration Δ*t*. In Figure 2e, the averaged step size is 0.25 nm (discussion of the spatial resolution of the image analysis method is provided in Note S1, Supporting Information).

To further investigate the pulse‐wise nature of OTE motors, the velocity maxima are extracted from a number of measurements and are plotted in Figure 2f, which exhibit linear relation with the repetition rate and correspond to a maximum step size of ≈0.6 nm. The data points within 5000 Hz are acquired by using the microscopic camera (cf. Figure 2e). Owing to the limited temporal resolution of the recording device, the data point marked by a red contour is obtained by synchronizing the motion of the gold plate with the electric moving stage, the latter set to translate at a fixed speed of 11 µm s^−1^ (see Movie S3, Supporting Information). Initiation of this synchronization should be under the circumstance that relative stillness is maintained between the motor's centroid and the light spot center in a configuration close to optimum, so that the gold plate could constantly travel at the maximum speed to keep pace with the moving stage (or the moving light spot when the stage is set as the reference frame). Note that a moving velocity exceeding 10 µm s^−1^ or 2.5 times the body length per second is highly remarkable in comparison to fluidic‐domain optical manipulation or even movements of macroscale motors. While maintaining a high spatial resolution by virtue of the pulse‐wise motion, the moving velocity of OTE actuators can be further enhanced by correspondingly increasing the repetition rate (up to 100 kHz) of the input laser.

For quantitative analysis of the motion dynamics as in Figure 2, gold plates that display regular geometries and have smooth planar surfaces are favored over layered materials. Considering that the image analysis is conducted along the horizontal plane, nonuniformity along the material thickness direction might compromise its accuracy, as it provides an extra source of asymmetry that could not be captured by optical lenses or the charge coupled device. For layered materials, implementation of the directional bias also leads to the centroid locomotion toward the light spot, whereas at the end of each individual motion step, the apparent centroid may not closely approximate the center of the light spot, owing to the existence of contact asymmetry and interlayer nonuniformity that are inevitably introduced in the mechanical exfoliation process. Despite this, omnidirectional navigation can be achieved on layered materials, which indicates the triumph of absorption asymmetry over small perturbations such as contact imperfections, and more importantly, the general applicability of the directed OTE actuation on materials featuring van der Waals contacts with planar solid substrates.

### 2.2 Actuation Mechanism

**Figure 3:**
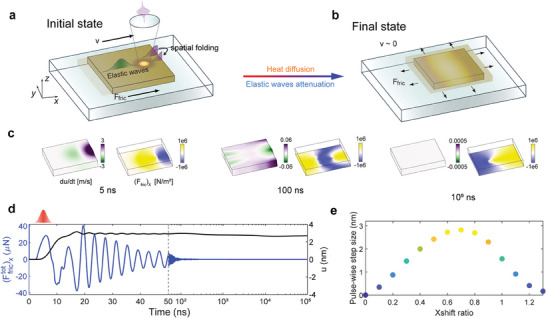
Mechanism of the OTE motion enabled by illumination asymmetry. a) Sketch of the initial state in which the decisive dynamics, featuring photothermally excited elastic waves and centroid motion driven by the induced friction force, occur within tens of nanoseconds. b) Sketch of the final state featuring an anchored centroid and a static thermal response (thermal contraction). c) Profiles of the *x*‐components of the deformation velocity (d*u*/d*t*) and contact friction (*F*_fric_)_x_ at different time following the pulse injection (at 5 ns). Evolution of d*u*/d*t* depicts the dynamics of elastic waves, and that of (*F*_fric_)_x_ conveys superimposed information of both the elastic wave dynamics and the static thermal response. d) Simulated centroid displacement and the total friction force exerted on the motor as functions of time. To clearly unveil both the transient dynamics of elastic waves and the diffusive thermal response according to their respective time scales (demarcated by the gray dashed line), the left part (*t*  ≤  50 ns) of the plot displays linear time scale and the right part (*t*  >  50 ns) displays logarithmic time scale. e) Simulated pulse‐wise step size variation with the level of deviation of the light spot. The Xshift ratio is defined as the ratio between the distance from the motor's centroid to the light spot and half the length of the motor. The optimum configuration corresponds to the maximum pulse‐wise motion step. In (c–e), coupled thermal‐elastic‐friction simulations are performed to study the OTE motion of a square‐shaped gold plate (side length 10 µm, thickness 100 nm) on a glass substrate. A single shot of light pulse (pulse duration 2 ns; single pulse energy 100 nJ; 1/*e* diameter of the Gaussian light spot 4.3 µm) is directed at a distance (4 µm) to the right away from the plate's centroid while coinciding with one of its axes of symmetry (parallel to the *X*‐axis). The shear strength of the friction force is τ_fric_ = 10^6^ N m^−2^.

To provide more theoretical insights of the OTE technique, numerical simulations are performed on a square‐shaped gold plate (simulation settings are provided in Note S3, Supporting Information). In the OTE motion, the decisive dynamics take place within the first tens of nanoseconds after the injection of the light pulse, which is characterized by the intense transient interplay between the photothermally excited elastic waves and the induced friction force (see **Figure** **3**a and Note S2, Supporting Information). Through elastic wave attenuation and heat diffusion, the OTE deformation gradually homogenizes, ultimately returning the entire system to a stabilized steady state (Figure 3b).

The friction force as the primary external force is induced by the excited elastic waves and contributes to the translation of the center of mass (centroid) of a motor. Specifically, after absorption of a light pulse by the motor, the temperature within the irradiated region quickly rises in a few nanoseconds (see Note S4, Supporting Information), which excites pulsed lattice vibrations, i.e., elastic waves. As the elastic waves propagate through individual portions of the motor, they intend to drive the motion of the latter, which is however resisted by the induced opposing friction force exerted on the van der Waals contacting surface. The friction force follows the elastic waves like a shadow, and their spatial profiles coincide with each other. Given this understanding, considering that the elastic waves carry deformation velocities in direction parallel to the *X*‐axis (d*u*/d*t*), the total induced friction force can be intuitively expressed as:

(1) $${\left(F_{\mathrm{fric}}^{\mathrm{tot}}\right)}_{x}=\tau_{\mathrm{fric}}\left(A_{-}-A_{+}\right)$$

where τ_fric_ denotes the shear strength of the friction force (≈ 10^6^–10^8^ N m^−2^),^[^ ^15^, ^16^, ^17^, ^18^ ^]^ and *A*_±_ is the area of the part of the contact surface on which the elastic waves render the motion in the $\pm \hat{x}$ direction. Apparently, to enable the centroid motion with a non‐vanishing ${\left(F_{\mathrm{fric}}^{\mathrm{tot}}\right)}_{x}$ from the perspective of Newton's law of motion, the key is to generate spatial non‐parity of the elastic waves (i.e., ensuring the inequality between *A*_+_ and *A*_−_).

The off‐centroid light illumination is introduced to trigger this needed spatial non‐parity of the elastic waves. As is sketched in Figure 3a, two groups of oppositely propagating elastic waves are excited from the irradiated region. The elastic waves that carry displacements aligned with their initial propagation directions are responsible for providing the biased motion, which signify as longitudinal acoustic waves. Elastic waves across a wide frequency span can be photothermally excited by the nanosecond pulsed laser,^[^ ^30^ ^]^ and it is their combined longitudinal components that determine the plate's motion. The deviation of the light spot center from the motor's centroid results in a scenario where one group of the elastic waves—initially traveling toward the short side (demarcated by the light spot)—outpaces the other group to undergo edge reflection, which is manifested as the spatial folding of the elastic wave profile, while the other group propagates undisturbed (cf. Figure 3a). Consequently, an imbalance is produced in the occupation areas of the two groups of said waves, embodied as the asymmetric distribution of the deformation velocity (d*u*/d*t*), which then induce an opposing profile of the friction force (the left panel in Figure 3c). Owing to its spatial non‐parity, the overall friction force exerted on the motor is biased and points from the motor's centroid to the light spot, fueling the initial acceleration of the motor's centroid in the same direction. The subsequent edge reflections in turn from both edges lead to oscillatory sign reversal of the friction force, thus decelerating the motion. Meanwhile, the elastic waves undergo strong attenuation by the impeding friction force exerted across the contact surface and through other loss channels (e.g., material defects), along with which the friction force evolves accordingly, as exhibited by the middle and right panels in Figure 3c (see Notes S2 and S4, Supporting Information for more details about the involved dynamics).

The simulated temporal variation of the centroid displacement (black solid line) and the total friction force ${\left(F_{\mathrm{fric}}^{\mathrm{to}t}\right)}_{x}$ (blue solid line) support above discussions (Figure 3d). Specifically, the initialization and termination of the centroid displacement synchronizes the steep ascent and descent of the total friction force. Their primary dynamics both terminate at an early stage (around 50 ns) and then maintain almost constant values throughout the remaining time. Notably, the centroid displacement is directed from the gold plate's centroid to the light spot center (i.e., +$\hat{x}$ direction), agreeing with the experimental observation. On the other hand, the driving force ${\left(F_{\mathrm{fric}}^{\mathrm{tot}}\right)}_{x}$ initially aligns with the centroid motion and subsequently exhibits fading oscillations due to edge reflections, wave attenuation as explained before.

The actuation mechanism discussed above sheds light on the comprehension of the experimental observations. First, the existence of an optimum configuration between the light spot and the centroid of the OTE motor (refer back to Figure 2d) mainly stems from the trade‐off between the magnitude of the directional bias and that of the total light absorption (see Figure 3e). In particular, once the light spot is placed at the close proximity to the edge of micro‐actuators, a portion of the injected light energy would fall outside of the actuator and not be received, which lead to a reduced momentum of excited elastic waves and hence the reduced pulse‐wise step size. A secondary reason accounting for this phenomenon is that, between the two groups of counter‐propagating waves, one group of elastic waves would instantly undergo reflection and propagate in the same direction as the other group without considerable delay, which compromises both the temporal duration and the degree of the spatial non‐parity. Second, the observation that OTE systems are capable of supporting high repetition frequencies of light (≈MHz) is because of the rapid attenuation of elastic waves. By virtue of the van der Waals contact, the oscillatory dynamics of elastic waves cease within tens of nanoseconds together with the centroid motion of the motor (Figure 3d), which are unrestricted by the slow progression of heat transfer (see more explanations in Note S4, Supporting Information). This makes high‐speed transportation achievable, on the condition that the actuation system can endure a certain level of heat accumulation (more discussion on this matter can be found in Note S5, Supporting Information).

### 2.3 Reconfigurable and High‐Resolution OTE Assembly

**Figure 4:**
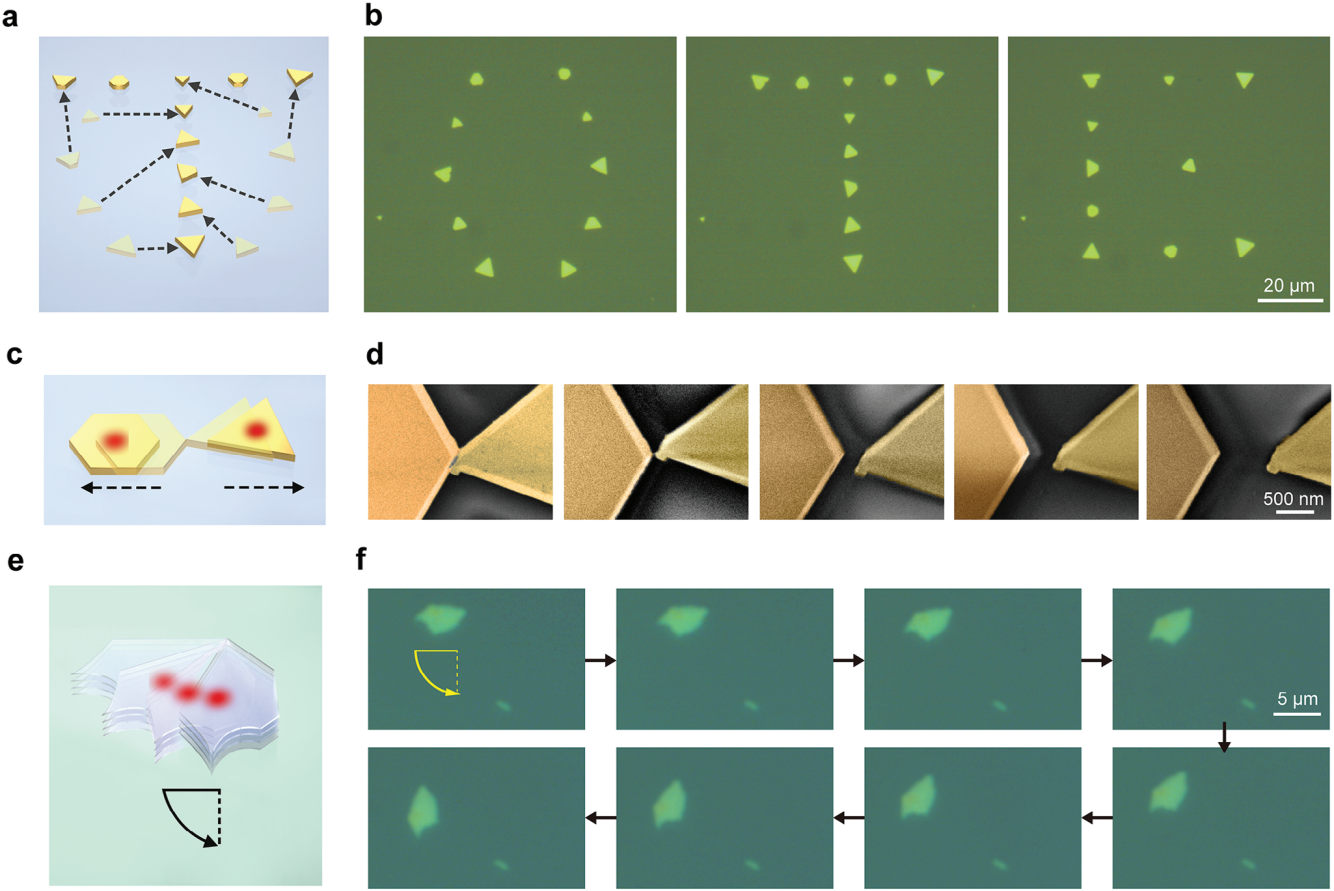
Reconfigurable and high‐resolution OTE assembly. a) Schematic diagram of the rewritable optical assembly on solid surfaces. b) Optical microscopic images of the letters “O,” “T,” and “E” constructed by actuating ten different gold plates on a glass substrate. c) Schematic and d) false color SEM images showing adjustment of the plasmonic gap distance between two gold plates using the OTE technique. e) Schematic and f) optical microscopic images showing the controlled in‐plane rotation of an exfoliated MoS_2_ flake on an indium tin oxide substrate.

The OTE actuation technique provides an unprecedented capability for precise control of micro‐objects on solid substrates, which can be harnessed to conduct all‐optical assembly and disassembly, as illustrated in **Figure** **4**a. Figure 4b consecutively shows letters “O,” “T,” and “E” assembled by gold plates of different size and shape on a quartz glass substrate. The same ten pieces of gold plates are employed as basic building blocks when conducting the optical assembly, and the assembled letters all reside on the same portion of the substrate. To begin with, the ten gold plates are transported from surrounding locations to the selected spots using the OTE technique. The average range of transportation is around 400 µm. To construct the first letter “O,” a beam of pulsed laser is focused and, with coordination of the translation stage, picks up target gold plates one by one and sequentially moves them to their destined positions. Further construction of the other two letters requires the previously assembled letter to be disassembled and that the gold plates be redirected to new sites (Figure 4a).

To further examine the spatial resolution of the OTE technique, a plasmonic gap is built between a hexagonal and triangle gold plates utilizing a similar procedure as discussed above (Figure 4c). The nanoscale gap dimension is adjusted by controlling the number of input pulses as well as the relative position between the light spot center and the actuator's centroid. Figure 4d shows a series of SEM images of the plasmonic gap. Initially, the two plates are brought into close proximity (gap distance = 0) using the OTE actuation technique, and then the gap is widened gradually by incrementally shifting one plate relative to the other (this process is also displayed in Figure S5, Supporting Information). Defining the gap distance as the closest distance between the two plates, as observed in Figure 4d from left to right, the respective measurements are 0, 15, 160, 310, and 610 nm. The manipulation capability showcased here corresponds to a spatial resolution at nanoscale, which can be further pushed forward through finer control over the input pulse trains or the use of in situ characterization techniques with comparable resolution.

Aside from the centroid translation, changes of orientations can be induced on OTE motors, which correspond to the in‐plane rotation (also observed in Figure 1e,f). It occurs as the result of a rotational symmetry breaking configuration, i.e., when the light spot center deviates from the axes of symmetry of the motor (more explanations are provided in Note S6, Supporting Information). Most of the time, in‐plane rotation comes as a byproduct of the centroid translation, considering that various factors, such as the misalignment of the optical path, an elliptical light spot, or the intrinsic chirality of the target actuator, could break the rotational symmetry of the actuating system. Nevertheless, this degree‐of‐freedom of motion can still be precisely modulated by exploiting the pulse‐wise nature of the OTE actuation. To demonstrate this capability, a small flake of exfoliated MoS_2_ is controlled to rotate anticlockwise on an indium tin oxide substrate (Figure 4e). Figure 4f sequentially display optical microscopic images of the target flake with different orientations. Since the lack of rotational symmetry, which is characteristic of exfoliated materials with irregular geometry, inevitably produces the symmetry breaking necessary for translational motions, the flake also exhibits minor centroid displacements. Defining the rotational angle as the angle between the present and initial orientations, from left to right in Figure 4f, the respective measurements are 0°, 5°, 16°, 27°, 42°, 48°, 58°, and 77°.

### 2.4 Application on Integrated Devices

In recent years, on‐chip integration of microscopic materials has been a prominent research topic, in which the appended materials serve as probes that detect or modulate signals transmitted in opto‐electronic devices.^[^ ^14^, ^37^, ^38^, ^39^, ^40^ ^]^ Targeted transfer of materials is conducted using wet etching,^[^ ^41^, ^42^ ^]^ polymer‐supported stamping^[^ ^39^, ^43^ ^]^ or thin film patterning,^[^ ^37^, ^42^ ^]^ etc., which typically involve a series of chemical treatments, complex nanopatterning and alignment procedures. In spite of its precision and non‐contact characteristics, the method of optical manipulation is always precluded as candidates for material transfer, because it requires fluidic environments and high field intensity that are either incompatible with standard chip testing systems or damaging to the silicon‐on‐insulator (SOI) substrates. Herein, we demonstrate the exploitation of the OTE approach for material placement on integrated micro‐ring resonators (MRRs) with high‐precision, minimum contamination and reconfigurability.

**Figure 5:**
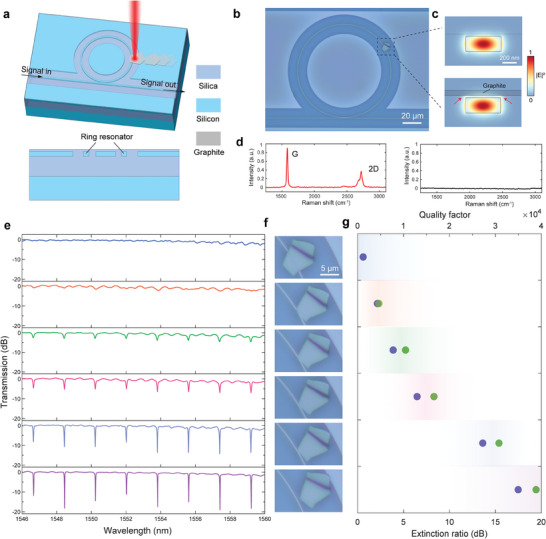
Precise OTE manipulation on integrated optical devices. a) 3D schematic (upper panel) and cross‐sectional view (lower panel) of the planarized MRR device. b) Optical microscopic image of the planarized MRR device with a fragment of graphite appended on top of it. The graphite flake is transported from a position away from the device using the OTE actuation approach. c) TE‐polarized modal profiles of the MRR waveguide at 1550 nm wavelength with (lower panel) and without (upper panel) the transferred graphite. The thickness of the graphite is measured to be 73 nm using an atomic force microscope. d) Raman spectra measured upon the positioning of the graphite (left panel) and after it is moved away (right panel). e) Transmission spectra of the MRR device with controlled positioning of the appended fragment of graphite as the elementary building block. f) Optical microscopic images exhibiting the relative position between the graphite flake and the core region of MRR. The images are aligned in a column in correspondence with the vertically arranged subfigures in (e) and (g). g) Extinction ratio and quality factor of the MRR at the resonant wavelength near 1553.8 nm with controlled positioning of the graphite flake. The green and purple symbols are the quality factor and extinction ratio, respectively, extracted from the measured spectra in (e). Note that at the “off” state, due to the resonant dips being largely submerged in the background, the value of the quality factor could not be obtained. The average power and repetition rate of the light source utilized in transportation and placement of the graphite flake is 22 µW  and 100 Hz, respectively.

**Figure** **5**a schematically shows the MRR device used in experiments. The fabrication of MRR follows standard complementary‐metal‐oxide‐semiconductor (CMOS) processes, which finalizes with an extra chemical mechanical polishing step to produce a nearly flat surface. Mechanically exfoliated fragments of pyrolytic graphite are transferred onto the substrate using the polydimethylsiloxane (PDMS)‐assisted stamping method.^[^ ^44^ ^]^ Note that the PDMS stamp did not touch the MRR device; instead, the stamp was purposedly placed outside the region of MRR to avoid contamination caused by adhesion of unwanted materials (see more fabrication details in Figure S6, Supporting Information). After that, a selected flake of graphite is transported to approach the MRR by employing the OTE technique (Figure 5b). The range of transportation is a few millimeters, corresponding to the size of the stamp. Figure 5c depicts modal profiles of the fundamental TE‐polarized modes in the MRR waveguide with and without the appended graphite cladding layer, revealing interactions between the graphite and the evanescent light field (indicated by red arrows). Raman spectra are collected both upon the placement of the graphite on top of the MRR and subsequent to its removal navigated by the pulsed laser, as shown in Figure 5d. The peaks emerging at 1583 and 2710  cm^−1 ^ are fingerprints of multilayer graphite, the disappearance of which indicates that no residue substances are left after the material's removal (right panel in Figure 5d).

Upon adjustment of the concrete position of the graphite flake relative to the waveguide, the transmission spectrum of the target MRR is modulated accordingly, as shown in Figure 5e. For reference, Figure 5f displays optical microscopic images of the graphite incorporated on the MRR device, suggesting the relative position between the graphite and the waveguide's core region, which is aligned with the measured transmission spectra in Figure 5e from top to bottom. Figure 5g shows the extinction ratio and quality factor of the MRR calculated at the resonance wavelength near 1553.8 nm, similarly aligned to Figure 5f to illustrate the correspondence. Specifically, when the flake is placed away from the core region of the ring resonator (bottom panels of Figure 5e–g), no modulation occurs in relation to the bare device without appended materials. With light passing through the resonator undisturbed by the graphite, it is marked as the “on” state. In contrast, all resonant dips disappear when there is a full coverage of the graphite on top of the silicon core (top panels of Figure 5e–g). Under this circumstance, no light passes through the resonator due to the significantly enhanced loss, turning the waveguide‐MRR coupling from critical‐ to the under‐coupling regime (coupling rate ≪ damping rate), which effectively marks the “off” state of the MRR. By moving the graphite away from the core region, the “off” state can be fully reverted back to the original “on” state and vice versa, as the moderate light intensity of ≈50 W cm^−2^ employed in this experiment would not cause damages to the SOI substrate or the switch element, which is over four orders of magnitude lower than that required in optical tweezer experiments (10^6^–10^8^ W cm^−2^),^[^ ^45^, ^46^ ^]^ neither would there be residue contaminants after the removal. Intermediate states can be obtained by placing the graphite flake near the core region or partially covers it, and the extinction ratio and quality factor would experience varying degrees of reduction relative to those of the bare MRR (Figure 5g).

## 3 Discussion

We have reported an all‐optical technique for precise and omnidirectional manipulation of micro‐objects on planar solid surfaces, which are the most ubiquitous and general paradigms of the solid environment. While retaining the merits of conventional optical manipulation, such as high precision, the ability to support different modes of locomotion and a diversity of material systems, the proposed OTE actuation further expands its capability into non‐fluidic domains with a simpler experimental configuration, relaxed requirements for light power and focusing.

Based on the current experimental setups, the lateral dimension of the micro‐objects that can be manipulated using the OTE technique is in the range of 4–20 µm. To further push down the size limit, objectives with higher numerical apertures or a driving laser with a shorter wavelength should be employed to preserve the directional bias, i.e., the absorption asymmetry. On the other hand, when actuating an object with larger dimensions, a more loosened laser focusing is preferred to mitigate the local overheating. Specifically, laser focusing and the single pulse energy should be adjusted in a coordinated manner to ensure that the micro‐object can be actuated before being ablated by the locally injected heat, and the size limit can be expanded by exploring a wider parameter space.

The pulse‐wise locomotion pattern of the OTE actuation suggests a sub‐nanometer spatial accuracy equivalent to a single lattice distance, which is however not fully explored in this current work. In the future, when combined with more advanced characterization technologies or employed in physical systems that are sensitive to atomic‐scale movements, we believe that direct proof of the superior spatial resolution of the OTE technique can be provided. By offering a powerful tool to approach extreme physical limits at atomic scale, the OTE actuation could bring new opportunities in areas such as interface engineering, heterostructure superlattices, controlled‐range electron and hole tunneling, as well as for applied sciences including the development of optical and electronic modulators and signal switches.

## 4 Experimental Section

### Sample Preparation

The micro‐sized gold plates were chemically synthesized through an aniline‐assisted (C_6_H_7_N) route in a heated ethylene glycol [(CH_2_OH)_2_] solution of hydrogen tetrachloroaurate (HAuCl_4_·4H_2_O).^[^ ^47^ ^]^ The as‐prepared gold plates were single crystals with hexagonal, triangular and trapezoid (truncated triangular) shapes, the thickness and horizontal dimension of which typically fall within a range of a few tens or hundreds of nanometers, and a few micrometers to several tens of micrometers, respectively. The gold plates were precipitated from the initial solution of ethylene glycol and subsequently dispersed in ethanol (CH_3_CH_2_OH). Glass slides (soda‐lime glass, commercially purchased; quartz glass, Corning 7980) were immersed in the ethanol solution to facilitate the collection and deposition of the dispersed gold plates. After that, ethanol‐bathed sonication was performed on the glass slides to remove the gold plates with poor van der Waals contacts with the substrates, while retaining those with complete and intact contacts. Layered material nanosheets were obtained through mechanical exfoliation from bulk crystalline materials (highly orientated pyrolytic graphite; molybdenum disulfide crystals) and then transferred onto the target substrates (polished silicon, planarized SOI substrates and ITO substrate, respectively).

### Characterizations

Measurements of the height profile were conducted using atomic force microscopes (Bruker Dimension ICON; Asylum Research Cypher ES). Scanning electron microscope (Zeiss Crossbeam 500) was employed to characterize the horizontal morphology of samples with nanometer resolution. Measurements of the Raman spectra were carried out with a 532 nm excitation laser using a confocal microscopic system (WITec Alpha 300R). Cross‐sectional samples at the material–substrate interface were prepared using a dual beam scanning electron microscopy‐focused ion beam system (ThermoScientific Helios 5 UX), which were then characterized by the transmission electron microscope (ThermoScientific Spectra Ultra).

### OTE Actuation System

The employed pulsed laser works at the wavelength of 1030 nm, with a pulse duration of 2 ns. The laser is subject to external modulation. Its repetition rate can be tuned from 1 to 200 kHz, and the emission of light pulses (both the timing and the number of pulses) can be modulated by inputting triggering signals from waveform generators. Before the driving light is guided into the microscopic system, its single pulse energy can be adjusted by the attenuator. The infrared objectives (20X, NA = 0.40; 50X, NA = 0.42) in the microscopic system are from Mitutoyo. Characterization of the focused light spots is conducted using an infrared charge coupled device and is shown in Figure S1 (Supporting Information). The translation stage possesses a positioning accuracy of 100 nm, which carries the sample (material‐substrate system) to move in the horizontal plane and alters the relative position between the actuator and the light spot, while the position of the latter is fixed.

## Conflict of Interest

The authors declare no conflict of interest.

## Author Contributions

M.Q. led the whole research project with W.Y. Q.J. conceived the idea with W.Y. and M.Q. Q.J. worked on the experiments and collected the data. X.S. designed and built the optical drive system. R.T. and L.L. provided the MRR sample and conducted the transmission spectra measurements. W.T. contributed in material preparation. J.Z. and P.W. provided the gold plate samples. Q.J. and W.Y. performed theoretical calculations and analysis. W.Y. and M.Q. supervised the project. Q.J. and W.Y. wrote the manuscript with inputs from all authors.

## Supporting information

Supporting Information

Supplemental Movie 1

Supplemental Movie 2

Supplemental Movie 3

## Data Availability

The data that support the findings of this study are available from the corresponding author upon reasonable request.
