# Supplementary material for: Precise and Omnidirectional Opto‐Thermo‐Elastic Actuation in Van Der Waals Contacting Systems
Source: Adv Sci (Weinh). 2024 Aug 19;11(39):2401418. doi: 10.1002/advs.202401418 (PMC11497110; doi:10.1002/advs.202401418)
Supplement: Supplementary file 1 — Supporting Information [file ADVS-11-2401418-s002.pdf]

ADVANCED SCIENCE
Open Access

Supporting Information

for *Adv. Sci.*, DOI 10.1002/advs.202401418

Precise and Omnidirectional Opto-Thermo-Elastic Actuation in Van Der Waals Contacting Systems

Qiannan Jia, Renjie Tang, Xiaoyu Sun, Weiwei Tang, Lan Li, Jiajie Zhu, Pan Wang, Wei Yan and Min Qiu**

Supplementary Information for

Precise and omnidirectional opto-thermo-elastic actuation in van der Waals contacting systems

Qiannan Jia[1,2,3] , Renjie Tang[2,3], Xiaoyu Sun[2,3], Weiwei Tang[4], Lan Li[2,3,5], Jiajie Zhu[6], Pan Wang[6], Wei Yan[*,2,3], Min Qiu[*,2,3,5]

1 College of Information Science and Electronic Engineering, Zhejiang University, Hangzhou 310027, Zhejiang Province, China.
2 Key Laboratory of 3D Micro/Nano Fabrication and Characterization of Zhejiang Province, School of Engineering, Westlake University, Hangzhou 310024, Zhejiang Province, China.
3 Institute of Advanced Technology, Westlake Institute for Advanced Study, Hangzhou 310024, Zhejiang Province, China.
4 College of Physics and Optoelectronic Engineering, Hangzhou Institute for Advanced Study, University of Chinese Academy of Sciences, Hangzhou 310024, Zhejiang Province, China.
5 Westlake Institute for Optoelectronics, Hangzhou 311421, Zhejiang Province, China.
6 State Key Laboratory of Extreme Photonics and Instrumentation, College of Optical Science and Engineering, Zhejiang University, Hangzhou 310027, China.
**Correspondence: Wei Yan (wyanzju@gmail.com) or Min Qiu (qiumin@westlake.edu.cn)*

This PDF file includes:

Fig. S1: Characterization of the focused Gaussian light spots at the focal plane
Fig. S2: Characterization of the actuator-substrate system
Fig. S3: Dynamics of the OTE actuation in different gold plate samples
Fig. S4: Dynamic locomotion of a gold plate with an initial configuration of light spot close to optimum
Fig. S5: High-resolution adjustment of the plasmonic gap distance between two micro-gold plates
Fig. S6: Preparation of the graphite-integrated MRR
Fig. S7: Attenuation effect of the friction force
Fig. S8: Simulated dynamics of the OTE actuation
Fig. S9: Theoretical calculations incorporating the in-plane rotation in a rotational symmetry breaking configuration
Fig. S10: Time-evolution of the out-of-plane torque integrated on the contact surface
Supplementary Note 1: Image analysis method for measuring the center of mass motion (supplementing Fig. 2 in the main text)
Supplementary Note 2: Elastic wave perspective
Supplementary Note 3: Numerical simulation of the OTE actuation
Supplementary Note 4: Dynamics of the OTE actuation (supplementing Fig. 3 in the main text)
Supplementary Note 5: Discussion on the possibility of heat accumulation
Supplementary Note 6: Mechanism of the in-plane rotation enabled by breaking the rotational symmetry

Other Supplementary Materials for this manuscript include the following:

Movies S1-S3

Supplementary Figures

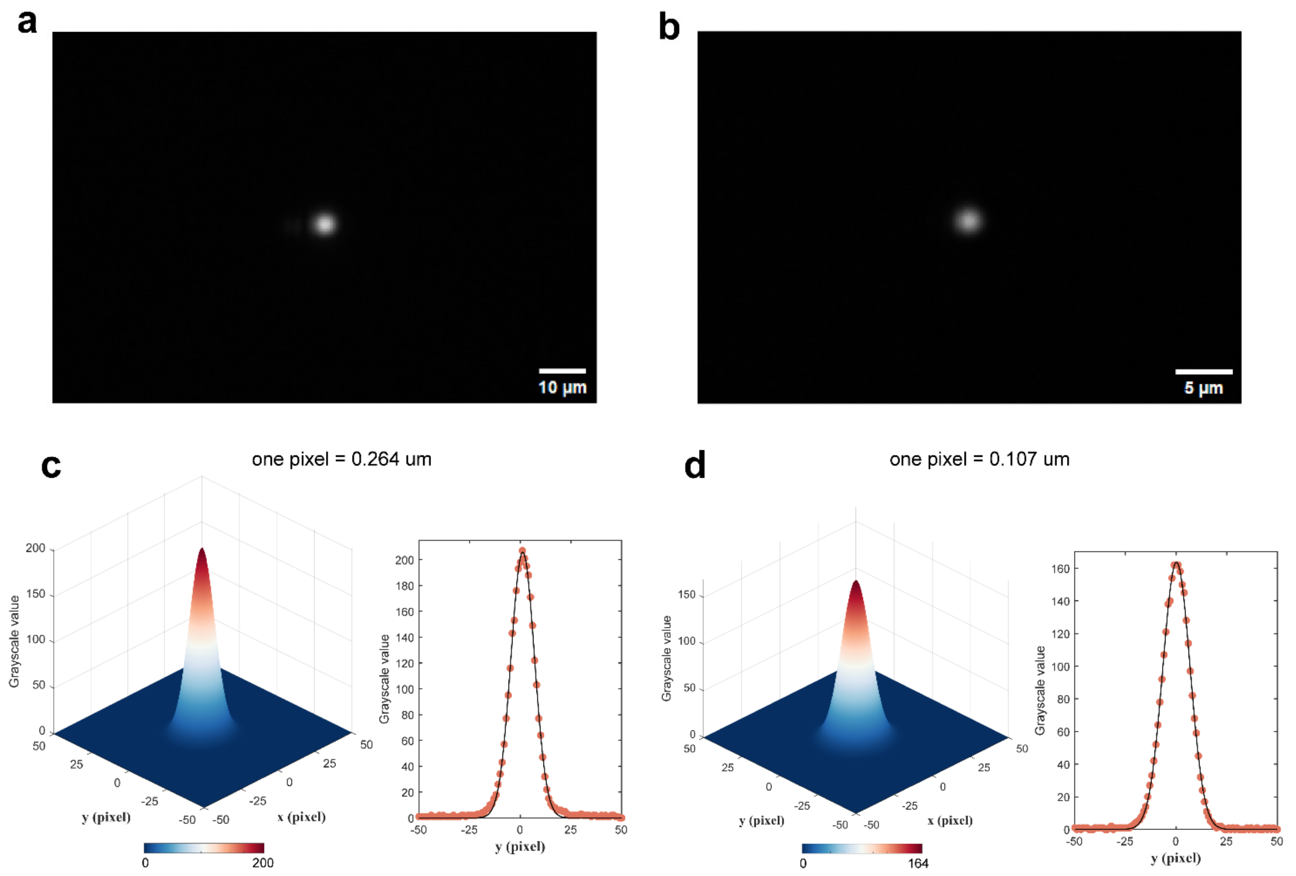

Figure S1. Characterization of the focused Gaussian light spots at the focal plane. Grayscale optical images of the focused light spots using **a.** a 20X objective (Mitutoyo, numerical aperture (NA) 0.40) and **b.** a 50X objective (Mitutoyo, NA 0.42). **c-d** are the Gaussian fitting results of the amplitude profile of the focused light spots in **a** and **b**, respectively. For convenience, the X and Y axes are scaled in pixels, with one pixel equal to 0.264 μm in **a** and **c**, and 0.107 μm in **b** and **d**. The fitting results suggest that the $1/e$ diameter of the Gaussian light spot is estimated to be 4.33 μm and 2.03 μm for the 20X and 50X objectives, respectively. The images are shot by an infrared charge coupled device (Thorlabs, near-infrared digital microscopic camera). The wavelength of the light source is 1030 nm.

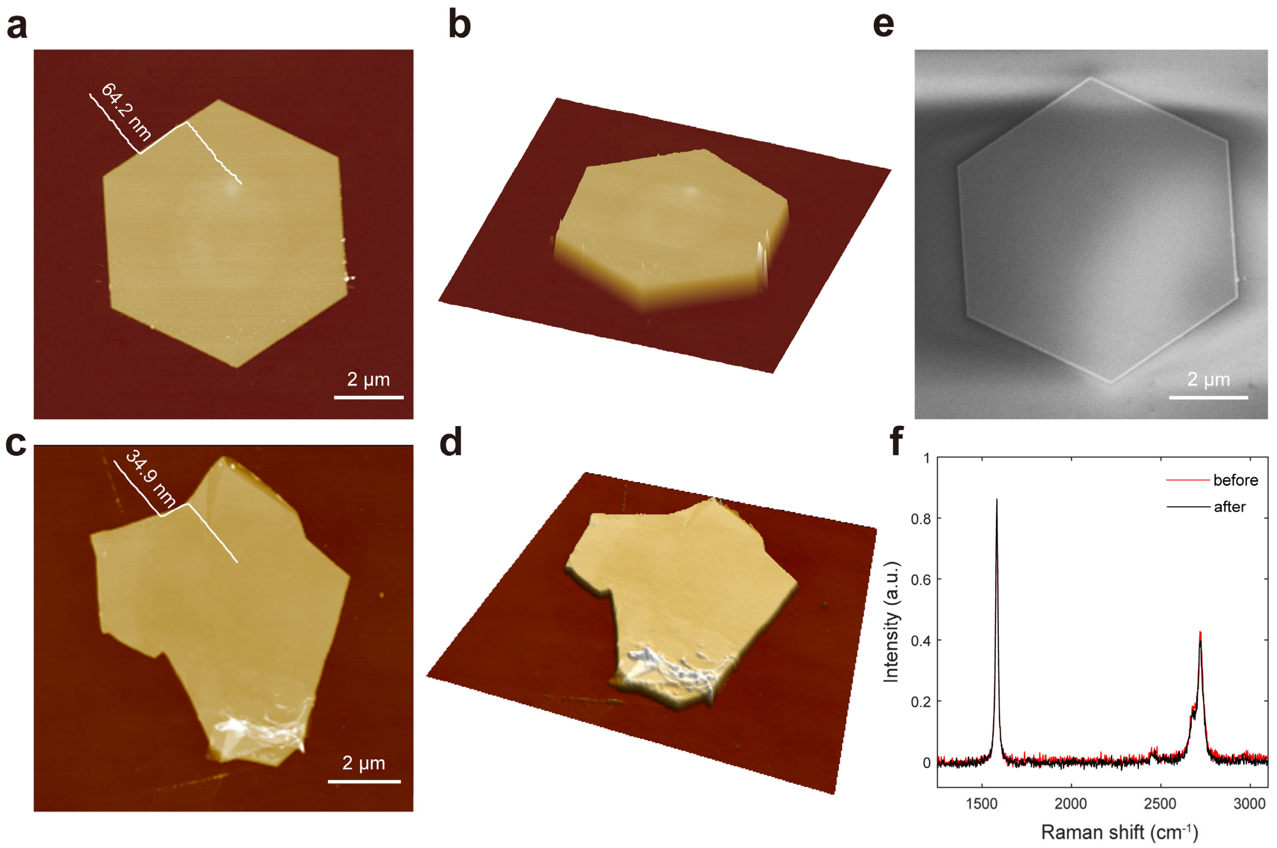

Figure S2. Characterization of the actuator-substrate system. a-d. 2D and 3D atomic force microscopic (AFM) images of (**a, b)** a gold plate on a quartz glass substrate and (**c, d)** an exfoliated pyrolytic graphite flake on a silicon substrate. The AFM images unveil smooth and planar contact morphologies between the gold plate and the silica glass, as well as between the exfoliated graphite and the underlying silicon substrate. **e.** Scanning electron microscopic image of the gold plate-quartz glass system after the OTE actuation. The anomalous contrast is due to the charging effect on an insulating surface. **f.** Raman spectra of the graphite flake before and after actuation, which exhibits no obvious alterations in either magnitude or the peak positions. The parameters for measuring the spectra are kept constant between the two measurements. Locomotion of the two actuators on corresponding planar solid substrates is displayed in Fig. 2 in the main text.

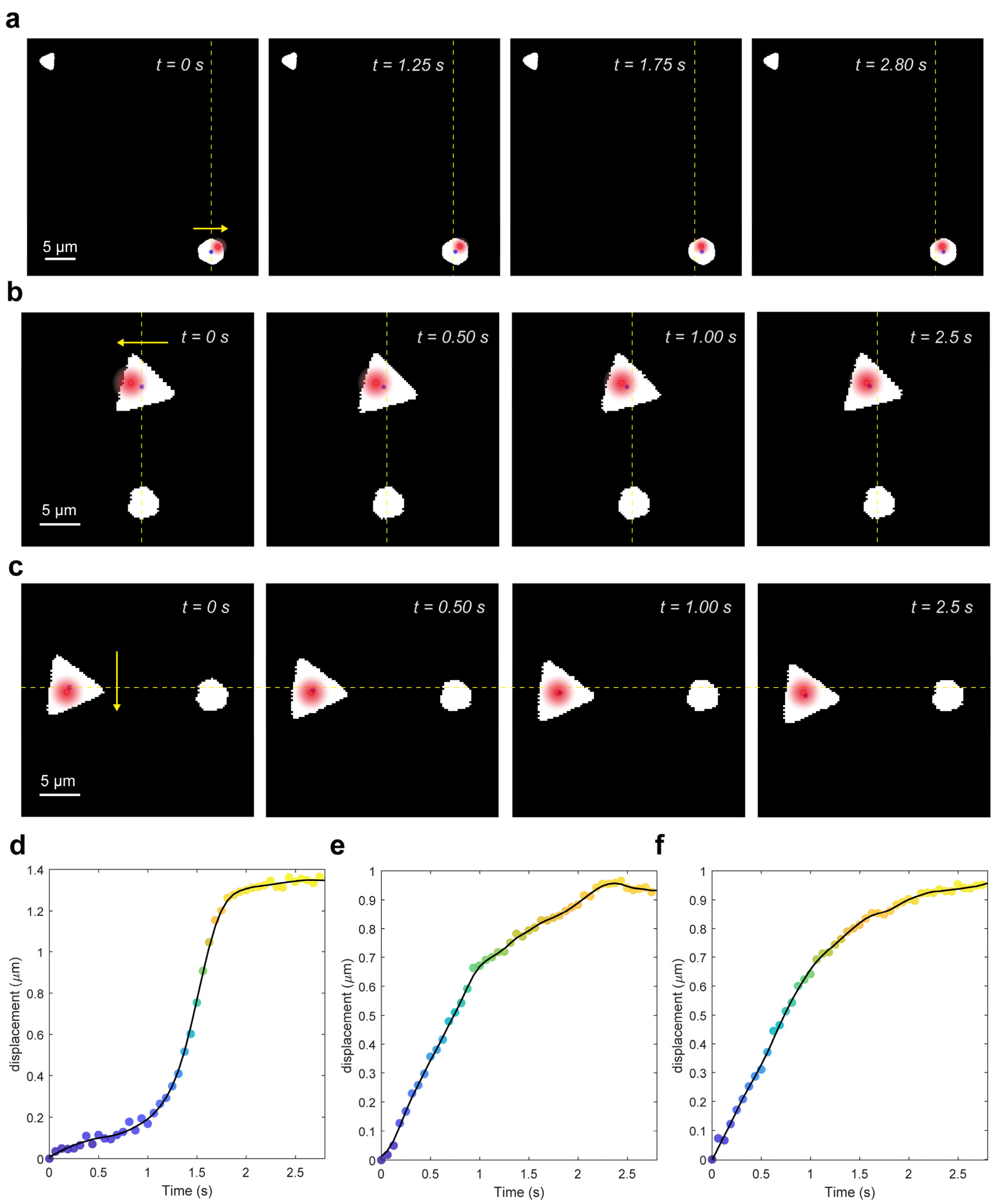

Figure S3. Dynamics of the OTE actuation in different gold plate samples. a. Successive optical images showing the centroid motion of a hexagonal gold plate along the horizontal direction and a triangular plate along the **b**. horizontal and **c.** vertical directions. The hexagonal gold plate is the same as that shown in Fig. 2 in the main text. The light spot is highlighted in red, and the centroids of the actuators are marked with blue asterisks. To facilitate the observation of the motors' movements,

dashed yellow lines are drawn perpendicular to the locomotion direction of the gold plates and through the initial position of their centroids. The $1/e$ diameter of the focused light spot, the laser power and repetition rate are 2.03 μm, 162.4 μW and 2 kHz, respectively, for the hexagonal gold plate, and 4.33 μm, 132 μW and 1 kHz, respectively, for the triangular plate. **d-f** are time-evolving centroid displacements of the processes shown in **a-c**, respectively.

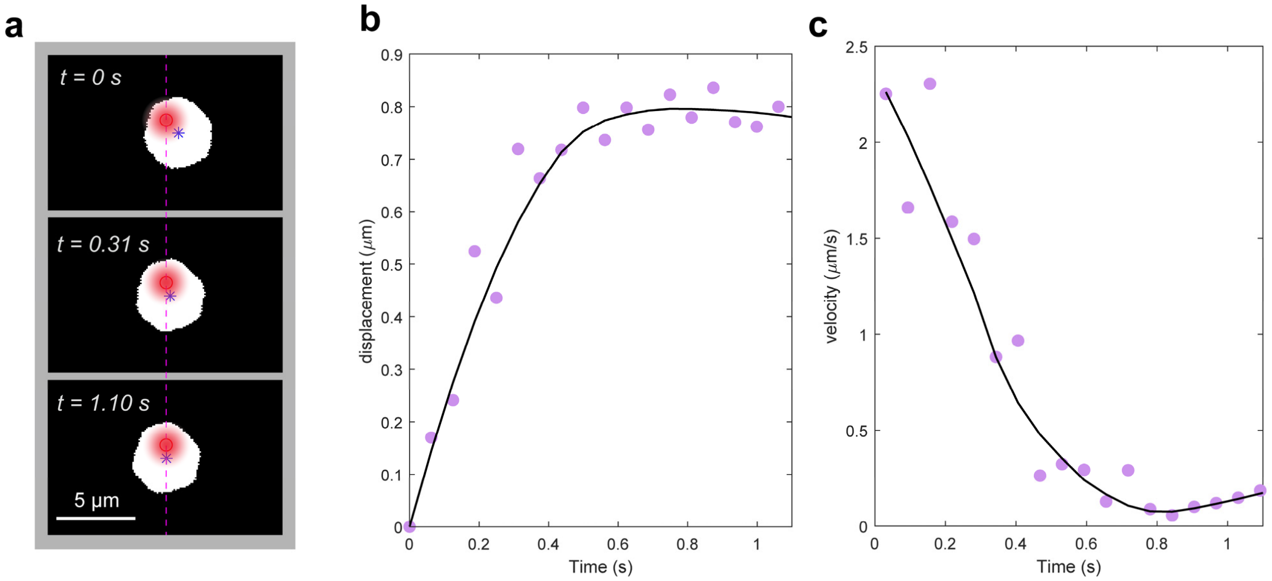

Figure S4. Dynamic locomotion of a gold plate with an initial configuration of light spot close to optimum. a. Snapshots showing the relative motion of a gold plate to the light spot. The plate commences its motion from a configuration associated with a high travelling speed (large pulse-wise step size). **b.** Captured displacement as a function of time of the target gold plate. **c.** Velocity evolution of the target gold plate deduced from **b.** The gold plate is the same as that shown in Fig. 2 in the main text. The employed laser power and repetition rate are 200 μW and 2500 Hz, respectively, with the laser beam being focused by the 50X objective.

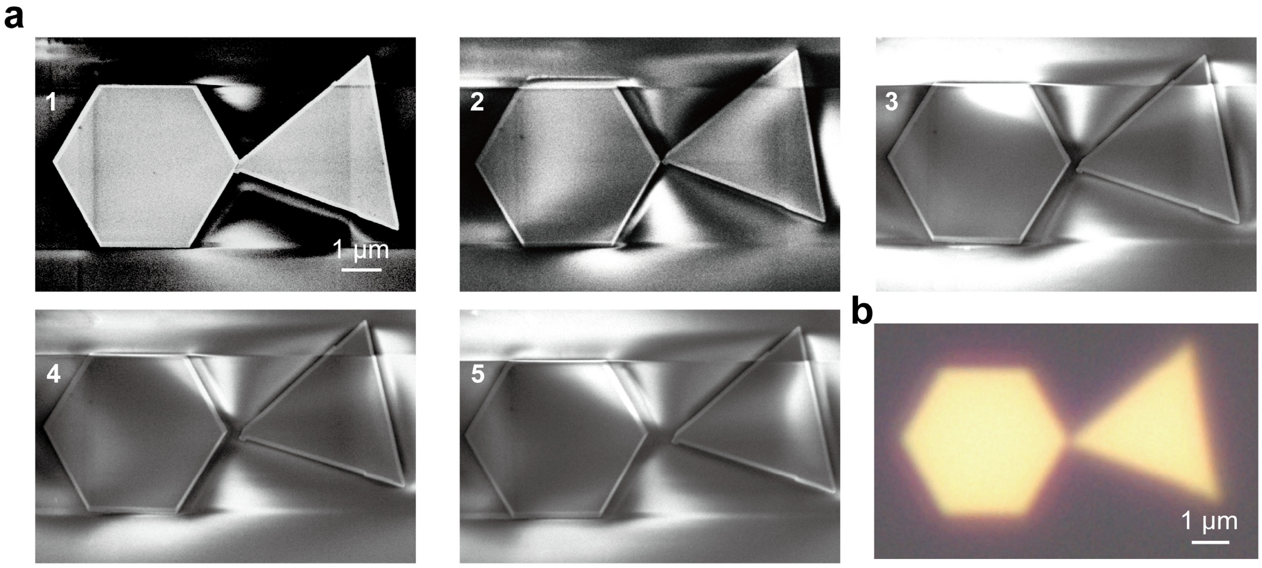

Figure S5. High-resolution adjustment of the plasmonic gap distance between two micro-gold plates. a. Sequential SEM images of two gold plates on a quartz glass substrate with controlled gap distance. Images from 1-5 correspond to Fig. 4d in the main text from left to right. The anomalous contrast is due to accumulation of electronic charges on a nonconductive substrate. Image distortions are largely ameliorated by using real-time drift correction methods while taking the SEM image, whereas those at the demarcation lines between the conductive gold material and insulating silica glass are hard to fully eliminate. **b.** Optical microscopic image of the two gold plates as in the first SEM image in **a.** The optical image is taken using a 100X objective (NA = 0.8). Comparison between the SEM images and optical image suggests that the nanoscale plasmonic gap has exceeded the observation capability of conventional optical systems, which, therefore, could not provide immediate feedback to assist the OTE manipulation process with nanoscale or even sub-nanometer resolution.

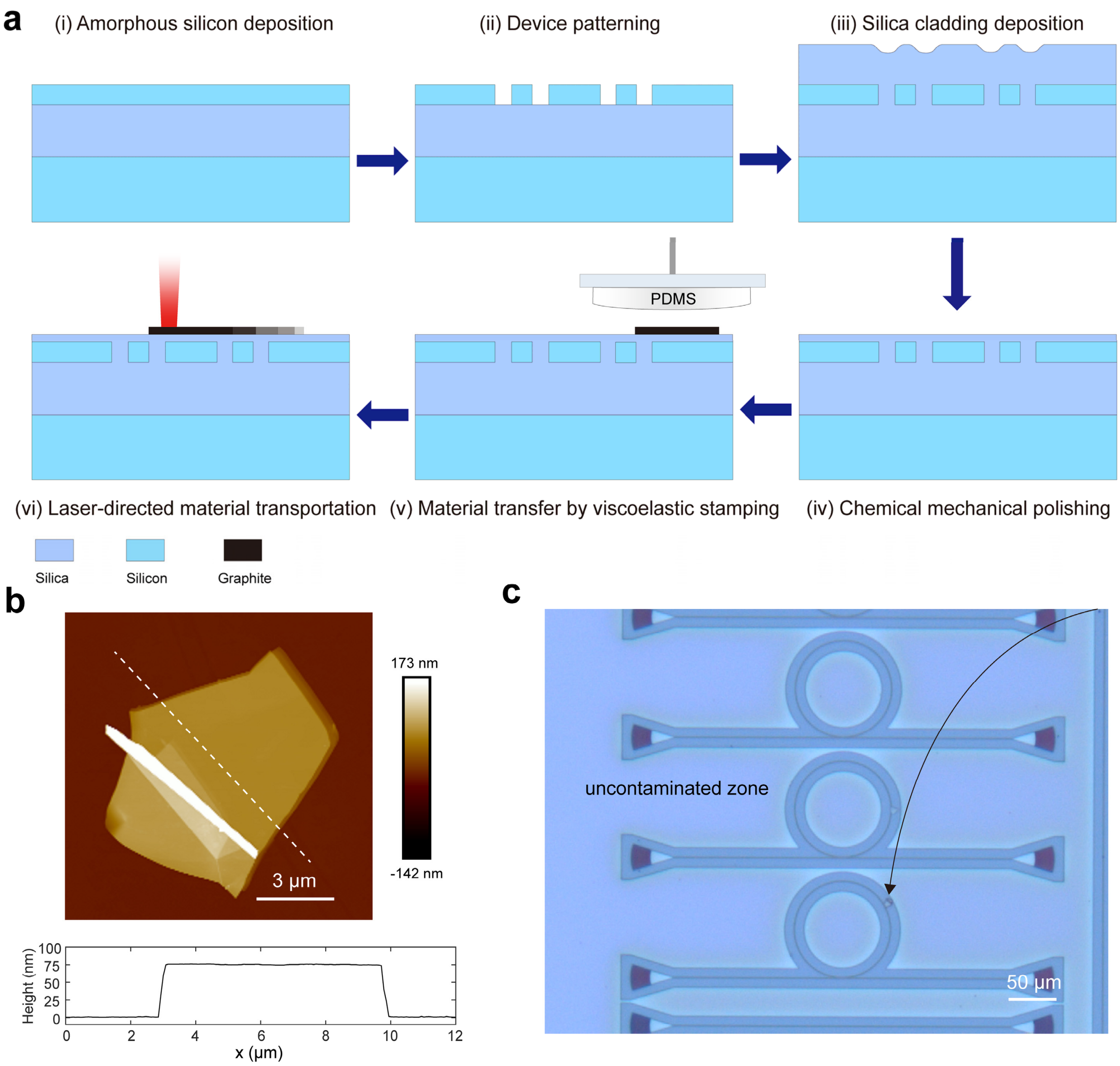

Figure S6. Preparation of the graphite-integrated MRR. a. Schematic illustrating the fabrication process flow of the MRR device. After chemical mechanical planarization, an upper cladding of silica is remained, which controls the strength of evanescent interaction between the appended graphite and the underlying waveguide. In experiments, the thickness of the upper cladding is 30 nm. In step (v), numerous fragments of exfoliated pyrolytic graphite are transferred onto the SOI substrate of the MRR via viscoelastic stamping, after which only a single graphite flake is picked out and driven to interface the MRR waveguide using the OTE actuation technique. The rest of the graphite flakes remain at their initial positions of deposition. **b.** AFM image of the selected graphite flake on the planarized substrate (upper panel) and the height profile of the white dashed cut line (lower panel). **c.** Optical microscopic image of the target MRR (at the bottom of the image) along with its neighboring optical devices on the same SOI chip. The arrow indicates the transportation trajectory of the selected flake from its initial position (outside the frame of the image) to the target spot. Owing to the viscoelastic stamping method that unexclusively transfer all the materials adhered on the stamp, the region where the stamp is placed

would be contaminated with unwanted materials. Supplemented with the OTE actuation approach, transportation and precise placement on the target device can be conducted exclusively on a single fragment of material, thereby avoiding introduction of additional contaminates to the region of immediate concern (marked as the uncontaminated zone).

Supplementary Note 1: Image analysis method for measuring the center of mass motion (supplementing Fig. 2 in the main text)

In the main text, the actuation dynamics was experimentally obtained by employing the image analysis method. The images for measuring the center of mass motion were captured by an optical microscopy, where the objectives have moderate numerical apertures (NA = 0.4, 0.42, see light spot characterization in Fig. S1). Despite the limited nominal resolution of the optical method, we managed to acquire the spatial resolution of the OTE actuation technique, defined as the time-averaged single step size of the center of mass motion, which is on the sub-nanometer scale. The discussion below clarifies how the image analysis approach can exceed its nominal resolution and reliably characterize the dynamics of the OTE actuation.

The image analysis approach we adopted in the main text relies on calculating the actuator's center of mass, rather than tracking the traveling trajectory of a specific spot or a feature landmark. The latter is limited by the nominal resolution of employed microscopies, which amounts to a few hundred nanometers for an optical microscopy with white light illumination. In contrast, to locate the center of mass of a micro-sized actuator, it is necessary to perform statistical averaging of the pixels in the binarized images (as shown in Fig. 2 in the main text). This leads to an significantly enhanced capability associated with resolving the centroid motion, or the collective motion of feature points on an actuator. Moreover, calculation of the averaged single pulse step, which we define as the spatial resolution of the OTE actuation, is based on measuring the traveling distance of the actuator's centroid over a train of several hundreds or thousands light pulses. The problem of determining the averaged single step size has been reduced to measuring the accumulated centroid motion divided by the number of input light pulses, akin to determining the thickness of a sheet of paper by measuring the thickness of a stack of several hundred sheets. The final precision of this method should rise with increased number of light pulses (or number of paper sheets).

By using the image analysis approach for tracking the centroid's motion, the measurement uncertainty mainly arises from the edge region, as the bulk region does not contain small features and is uniformly converted to white in the binarized image. Specifically, the area of the blurred edge region in the original optical image can be estimated to be $S_{blur} \sim \frac{\lambda}{2 \cdot NA} \cdot C$, where λ denotes the central wavelength of the illumination light source, NA denotes the numerical aperture of the employed objective, and C denotes the perimeter of the target actuator. This estimation is based on the observation that the blurry is caused by diffraction limit in microscopic systems, and the dimension of the blurry is on the same scale of the wavelength used in imaging. Consequently, the ratio of the blurred region relative to the total area of the target actuator is $\eta = S_{blur}/S_{total}$. Given that the measurement of the actuator's single pulse step is based on the accumulated motion of its centroid, the final measurement uncertainty

is calculated as $\sigma = (\eta \cdot \lambda)/(N \cdot 2NA)$, where $\eta \cdot \lambda/2 \cdot NA$ accounts for the uncertainty associated with a single measurement of the accumulated traveling distance, and N denotes the number of pulses. Taking 532 nm as the central wavelength in a white light illumination setup, assuming the NA of the objective is 0.4, and considering an actuator with a lateral dimension of 10 μm driven by 5000 light pulses, the measurement uncertainty of the image analysis method is about 0.035 nm (assuming the actuator is circular, and its uncertainty is calculated as $\frac{4 \cdot \pi \cdot d \cdot \lambda^2}{4 \cdot NA^2 \cdot \pi \cdot d^2 \cdot N}$, where d denotes the diameter/lateral dimension of the actuator). From the above discussion, we believe that the image analysis method sustains a high resolving capability, sufficient in determining the sub-nanometer precision of the OTE actuation defined as the average single pulse step size of the actuator's centroid.

Supplementary Note 2: Elastic wave perspective

The OTE actuation involves multi-physics-coupled processes. Given that the driving light is in the pulsed form, the process of heat generation inherits the impulsive nature of the energy input, and hence is able to photothermally excite elastic waves[1,2]. The excitation and propagation of elastic waves conform to the elastic wave equation. When disregarding the term introduced by the friction force, the elastic wave equation is expressed as below:

$$\rho\ddot{\boldsymbol{u}}(r,t)-\frac{E(1-\mu)}{(1-2\mu)(1+\mu)}\nabla\nabla\cdot\boldsymbol{u}(\boldsymbol{r},\boldsymbol{t})+\frac{E}{2(1+\mu)}\nabla\times\nabla\times\boldsymbol{u}(\boldsymbol{r},\boldsymbol{t})$$
$$=-\alpha_{th}\frac{E}{(1-2\mu)}\nabla(T(r,t)-T_0) \quad (1)$$

where $\boldsymbol{u}$ denotes the elastic displacement vector; ρ, E and μ are the mass density, Young's modulus and Poison's ratio of the acoustic media; α_{th} denotes the coefficient of thermal expansion; T denotes the temperature and T_0 is the ambient temperature. Specifically, the coupling between the pulsed laser and the elastic waves is mediated by transient thermal expansion of the irradiated media, which is introduced in the above equation as the "source" term. As a result of this source term, displacements $\boldsymbol{u}(\boldsymbol{r},t)$ are induced across the plate, which are variant with both time and space, manifesting as the opto-thermally induced elastic waves.

When taking into consideration the effect imposed by the friction force, another source term should be incorporated into the elastic wave equation:

$$\rho\ddot{\boldsymbol{u}}(r,t)-\frac{E(1-\mu)}{(1-2\mu)(1+\mu)}\nabla\nabla\cdot\boldsymbol{u}(\boldsymbol{r},\boldsymbol{t})+\frac{E}{2(1+\mu)}\nabla\times\nabla\times\boldsymbol{u}(\boldsymbol{r},\boldsymbol{t})$$
$$=-\alpha_{th}\frac{E}{(1-2\mu)}\nabla(T(\boldsymbol{r},t)-T_0)+\boldsymbol{f}_{fric}(\boldsymbol{r},t) \quad (2)$$

Compared to equation (1), the extra term $\boldsymbol{f}_{fric}(\boldsymbol{r},t)$ accounts for the friction force. For simplicity, equation (2) treats the friction force as the volume force, given that the OTE actuators are slab-like materials with small thickness. The effect of friction force rivals the contribution from the term of thermal expansion. As has been demonstrated in the main text, the friction force resists the relative motion between the actuator and the substrate, which functions to attenuate the elastic waves induced via transient thermal expansion. This explains why the oscillatory behavior of the displacement of OTE actuators ceases within tens of nanoseconds. Moreover, the friction force, as the sole external force exerted on the OTE actuator, is responsible for the directed motion of the actuator's centroid. Without friction force, thermal expansion alone is not able to trigger a net displacement of the overall actuator. The twofold effect of the friction force is clearly manifested in Fig. S7, where comparisons are made between simulation results with and without exertion of the friction force. Specifically, the oscillatory behavior of the actuator's deformation persists for significantly longer time for the simulation setting without the friction force, in contrast to the case with the friction force, where the

oscillation rapidly attenuates after light pulse injection. Moreover, the geometric centroid of the actuator, despite its intense oscillation, would return to its initial position after the elastic waves are naturally damped through conventional channels (e.g., material defects) without the friction force (Fig. S7b). In contrast, a net displacement would be retained in the case with the exertion of friction force (Fig. S7c).

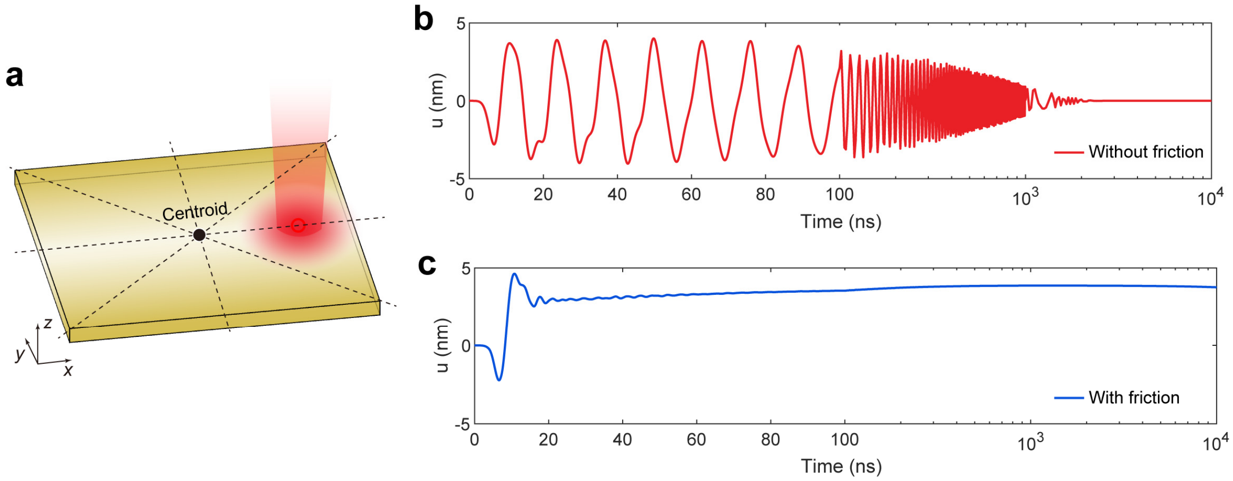

Figure S7. Attenuation effect of the friction force. a. Schematic of the simulation model. The deviation of the Gaussian light spot relative to the plate's centroid is along the positive *X*-axis direction. **b.** Time evolution of the displacement at the plate's geometric center (marked as the black dot in **a.**) without exertion of friction. **c.** Time evolution of the plate's geometric center with friction force. Note that the horizontal axes in the time evolution graphs are arranged in the way where the first 0-100 ns is in the linear scale and 100-10^4 ns is in the logarithmic scale. In the simulation model, the square plate has a lateral dimension of 10 μm, a thickness of 100 ns. The single pulse energy is 90 nJ, with a pulse width of 2 ns. The $1/e$ diameter of the Gaussian light spot is 4.33 μm. The light spot is 4 μm deviated from the plate's centroid. Apart from the friction force settings, the other simulation parameters are kept the same in acquiring results in **b** and **c.**

Supplementary Note 3: Numerical simulation of the OTE actuation

The OTE actuation technique involves an opto-thermo-mechanical coupled multiphysics process, which is simulated in time-dependent 3D models using the Comsol Multiphysics software. Specifically, heat transfer and solid mechanics modules are employed to account for the thermal field and the dynamics of elastic waves, respectively, and the coupling between the two modules is realized through the node of thermal expansion.

Participation of the electromagnetic field is simplified as the pulsed heat source in the heat transfer module, whose lateral profile should match the Gassuain profile of the light source. This simplification is based on the assumption that, considering the large disparity in dimension between the actuator (a few microns) and the light wavelength, their interactions primarily involve reflection and absorption, rather than wave scattering and interference. The latter would require more rigorous analysis using wave optics. Moreover, the pulsed nature of the light source should be embedded in the temporal evolution of the heat source. In general, the profile of the heat source is defined by two components: the spatial factor multiplied by the temporal factor. The magnitude of the heat source is proportional to the injected energy of a single light pulse and the absorptivity of the material of interest. Consequently, the volume heat source, which is superimposed on the geometry of the irradiated material in the simulation model, is expressed as:

$$Q(x,y,z,t) = \alpha \cdot E_{single} \cdot \frac{1}{(\pi \cdot w)(\sqrt{\pi} \cdot \tau)} \cdot e^{\frac{-[(x-x_0)^2+(y-y_0)^2]}{w^2}} \cdot \frac{1}{th} \cdot e^{\frac{-(t-t_0)^2}{\tau^2}} \quad (3)$$

where α denotes the material absorptivity, E_{single} denotes the single pulse energy; x_0 and y_0 are the deviation of the light spot center along *X*- and *Y*- axes relative to the origin *(0,0)*, respectively. The origin is set to be the centroid of the square actuator in the simulation; th denotes the thickness of the irradiated material; t_0 is the time delay of the pulse peak; w denotes the radius of the Gaussian light spot ($2w$ is the $1/e$ spot diameter), and τ denotes the half pulse width of the pulsed light (2τ denotes the pulse width). The term $\frac{1}{(\pi \cdot w)(\sqrt{\pi} \cdot \tau)}$ is to normalize the Gaussian functions in both the spatial and the temporal factors. Note that the spatial distribution of the heat source along the thickness direction (*Z*-axis) is treated to be uniform, given that the OTE actuators are slab-like materials with a thickness ranging from tens to hundreds of nanometers, comparable to the skin depth of the irradiated materials, and taking into account the rapid heat homogenization in this direction.

The governing equation in the heat transfer module is the heat transfer equation, primarily focusing on the heat diffusion process within the irradiated material. It specifically addresses the transfer of heat from the irradiated region to the surrounding areas, detailing the gradual homogenization of the material's temperature. The heat transfer equation is written as:

$$\rho C_p \frac{\partial T(r,t)}{\partial t} + \rho C_p \boldsymbol{v}(\boldsymbol{r},t) \cdot \nabla T(\boldsymbol{r},t) + \nabla \cdot \left(-k\nabla T(\boldsymbol{r},t)\right) = Q(\boldsymbol{r},t) \tag{4}$$

where $Q(\boldsymbol{r},t)$ denotes the volume heat source as in equation (3), in which $\boldsymbol{r}$ is the position vector; ρ, C_p and k denote the mass density, heat capacity and thermal conductivity of the material, respectively; $T(\boldsymbol{r},t)$ is the temporal-spatial varying temperature; $\boldsymbol{v}$ denotes the moving velocity of the finite element.

In the solid mechanics module, the linear elastic material model is employed, as the strain involved in the OTE actuation is small, and that the strain-stress relation should conform to the Hooke's law. The contact friction is introduced by adding a contact pair between the actuator and the substrate. The shear strength of the friction force is set to be in the order of $10^6\ N/m^2$, in accordance with the reported data at planar solid interfaces. The governing equation of the solid mechanics module is the elastic equation (equation (2)) with both source terms introduced by thermal expansion (through the thermal expansion multiphysics node) and the contact friction (through the contact pair node).

The simulation results exhibited in Fig. 3 in the main text and in the Supplementary Information are based on the irradiation of a single light pulse on a square gold plate attached to a silica substrate. The key parameters utilized in the simulation model are listed below:

1. Light source parameters:
   Time delay (t_0): 5 ns
   Pulse width (2τ): 2 ns

2. Material parameters:

	**Gold plate**	**Silica substrate**
**Density**, ρ $[kg/m^3]$	19300	2200
**Young's modulus**, E $[Pa]$	70×10^9	70×10^9
**Poisson's ratio**	0.44	0.17
**Heat capacity**, C_p $[J/(kg \cdot K)]$	120	730
**Thermal conductivity**, K $[W/(m \cdot K)]$	110	1.4
**Thermal expansion coefficient**, α_{th} $[1/K]$	31.5×10^{-6}	0.5×10^{-6}

Note that the simulation models depicted in Fig. 3 and Fig. S8 are identical, while the simulation model in Fig. S8 differs from the former in terms of the dimensions of the gold plate, the single pulse energy, and the diameter of the light spot. Please refer to the captions for more information.

Supplementary Note 4: Dynamics of the OTE actuation (supplementing Fig. 3 in the main text)

The elastic waves are photothermally excited on a transient time scale with the use of a nanosecond pulsed laser. In comparison, the process of heat transfer is characterized by slow heat diffusion and is lacking in transient features as opposed to the aforementioned elastic waves. Specifically, the elastic waves travel across the actuator at the sound speed of ~ 2000 m/s (for gold plates), which could complete several round trips within the micro-sized actuator in tens of nanoseconds. In the meantime, the elastic waves induce friction force that retroactively serves as a source of attenuation of the former. Owing to the strong damping effects of the friction force exerted through the van der Waals contacts, the elastic waves, together with their transient oscillatory features, would rapidly dissipate. The rest of the OTE dynamics after the first tens of nanoseconds would be dominated by the static thermal response: thermal expansion and contraction, which are regulated by the diffusive heat transfer.

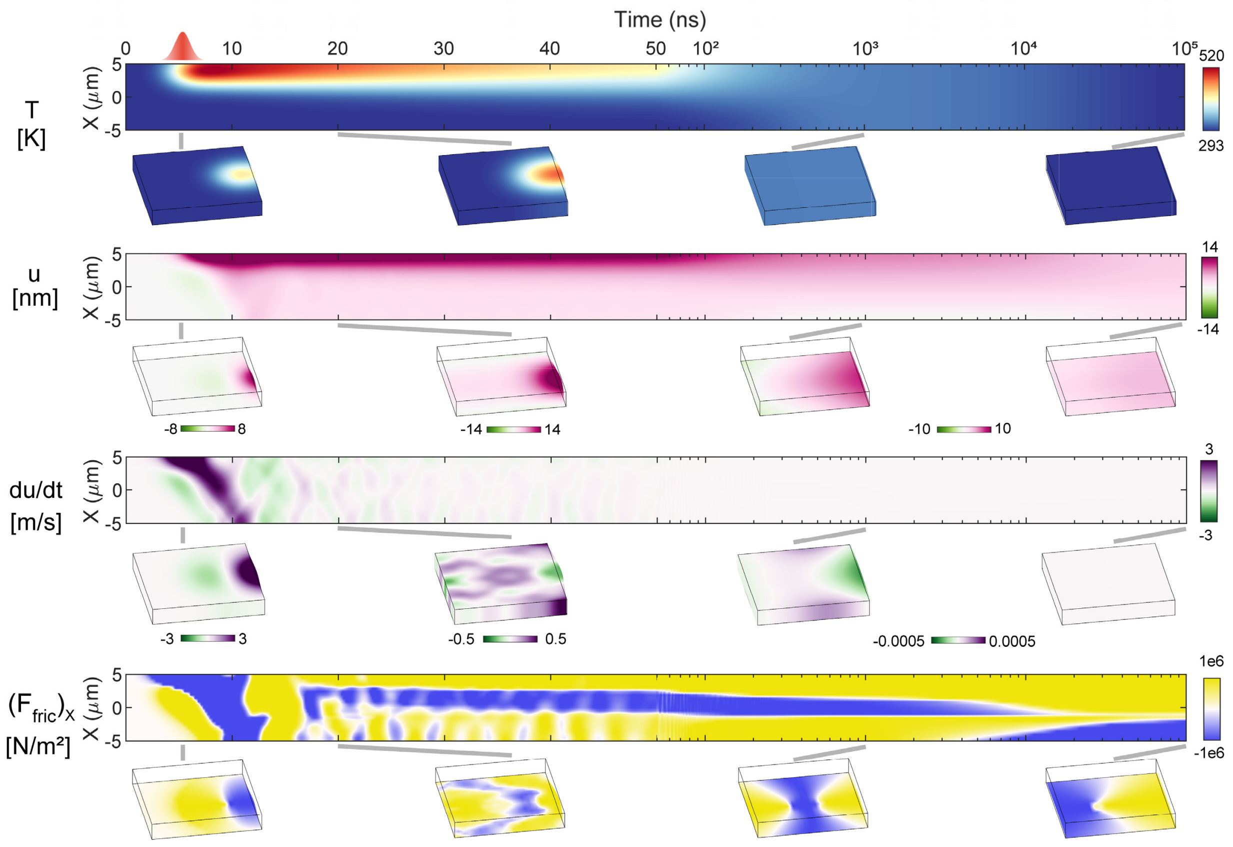

Figure S8. Simulated dynamics of the OTE actuation. From top to bottom are the evolving dynamics of temperature, *x*-components of the elastic displacement, deformation velocity and the contact friction along the axis of symmetry passing through the center of the contact surface. Profiles of respective physical quantities across the entire gold plate are probed at 5 ns, 20 ns, $1e^3$ ns and $1e^5$ ns and appended beneath each corresponding panel. To unveil both the transient dynamics of elastic

waves and the diffusive heat transfer, the left part ($t \leq 50$ ns) of the time-evolving profiles are in the linear time scale, while the right part ($t > 50$ ns) are in the logarithmic time scale.

To acquire a visual understanding of the involved dynamics, Fig. S8 displays the time-evolving profiles of temperature (*T*), x-components of the elastic displacement (u), deformation velocity (*du/dt*) and the contact friction $(F_{fric})_x$ probed along the *X*-axis parallel line across the contact surface (refer to Fig. 3a in the main text for the simulation model). Of the four quantities, the temporal variation of temperature portrays the dynamics of heat transfer and is associated with the static thermal response. The deformation velocity exhibiting zig-zag format reflects the propagation, reflection (spatial folding at the turning points of the zig-zag format) and attenuation of the non-static components of elastic waves. The elastic deformation and contact friction convey the superimposed information, encompassing both the static (thermal expansion, contraction and the as-induced friction that resist their trends) and oscillatory dynamics. A prominent spacetime separation between the elastic wave-related dynamics and the heat transfer process is unveiled, which is attributed to the huge disparity between the travelling speed of elastic waves and the rate of heat diffusion. The latter can be semi-quantitatively characterized by the diffusivity of the actuator material[3]. For gold plates possessing a thermal diffusivity $\alpha_{Au} \sim 10^{-4}\, m^2/s$, the time required for heat to travel a distance of $L = 10\ \mu m$ (the side length of the simulated square plate) is ~ $L^2/\alpha_{Au} \sim 1\ \mu s$. Hence, the critical period of the first tens of nanoseconds, during which the dynamics of elastic waves determine and drive the motion of OTE motors, can be decoupled from the slow progress of heat transfer and regarded as adiabatic[2], which explains the capability of OTE motors to accommodate ultra-high traveling speed (refer back to the main text).

Supplementary Note 5: Discussion on the possibility of heat accumulation

Figure S8 exhibits that the temperature of the irradiated gold plate could reach 520 K, which raises the question of potential heat accumulation in the OTE actuators. Depending on the concrete situation, discussion on this matter is provided below in three points:

1. Heat accumulation would not occur if the heat dissipates faster than the interval between neighboring light pulses, i.e., when the repetition rate of the pulsed laser is not too high (≫ 10 kHz). The upper panel in Fig. S8 in Supplementary Note 4 illustrates the heat dissipation process in the gold plate after the light pulse injection. As the figure suggests, intense heat localization occurs only in the first tens of nanoseconds, where a heated region concentrates near the illuminated area, reaching a temperature of ~ 500 K. The temperature across the plate homogenizes at ~ 1 μs, which is deduced from the thermal diffusivity of the material.

After 1 μs, the plate cools down uniformly, and approaching 10^5 ns, it returns to the room temperature. Given this simulation result, it is safe to argue that, as long as the next light pulse comes after 0.1 ms (10^5 ns, corresponding to a 10 kHz repetition rate) when the heat generated by the preceding pulse has dissipated adequately, the heating effects of neighboring pulses would not overlap. Hence, no heat accumulation would occur. When the repetition rate of the laser is way beyond 10 kHz, heat accumulation would occur, as the heat pump works faster than the heat dissipation channels, and the irradiated plate would experience pulse-wise temperature rise[4].

2. When the heat accumulation occurs mildly, i.e, when the repetition rate of the laser is slightly above 10 kHz, the plate maintains its structural integrity and continues to be actuated controllably.

As is demonstrated above, the heat homogenizes after ~ 1 μs. If the next light pulse comes more than 1 μs later than the injection of the first pulse (corresponding to a 1 MHz repetition rate), the plate would experience the same heat dissipation process but starting at an elevated temperature than the room temperature. Under this circumstance, the temperature of the plate tend to rise collectively and is more uniformly distributed across the plate (apart from the first tens of nanoseconds after the pulse injection), and its overall temperature would increase with time till a dynamic balance is established between the heat pump and the heat dissipation. In a previous study, the temperature threshold of laser ablation of gold micro-plates has been examined, which is around 500-750 K with steady-state heating (using the CW laser or the hot plate)[5]. The temperature maximum resulting from a single input pulse is 520 K in our simulation, which is below the ablation threshold, leaving ample room for the heat accumulation before the plate reaches thermal equilibrium. With the plate maintaining its structural integrity, it is highly likely that the plate's locomotion would not be affected by this accumulation of heat. This is evidenced by the steady locomotion of the plate with 20 kHz laser repletion rate (see the highlighted datapoint in Fig. 2f in the main text and Supplementary Movie 3).

However, when the repetition rate of the laser further increases, heat would be intensely accumulated within the irradiated region since it does not have sufficient time to dissipate to surrounding proportions. Severe thermal damage could occur that locally ablates the plate (as shown in the following optical microscopic image). As a result, the plate would lose its capability to be actuated using the OTE mechanism.

3. If there is none or only mild heat accumulation, the same microscopic object can be actuated infinite times, as the plate dose not undergo structural changes, and the OTE technique is translational invariant. When the heat accumulation is severe enough to induce laser ablation of the plate, it may no longer be capable to withstand repeated actuation. It would cease moving when it is damaged to the extent where its main structure framework is gone.

Supplementary Note 6: Mechanism of the in-plane rotation enabled by breaking the rotational symmetry.

Experimental results suggest that, while exhibiting translational motions with off-centroid illumination, OTE actuators also experience changes of orientations, as shown in Fig. 1 (**e**,**f**), 2 (**a**,**b**), 4 (**b,d,f**), and 5 (**f**) in the main text. More precisely, these changes of orientations correspond to a locomotion mode, which we summarize as the in-plane rotation of motors in the contacting plane around an axis perpendicular to the planar substrate. Similar to the philosophy of translational motions, to induce in-plane rotation of motors, a bias should exist along the azimuthal direction to break the rotational symmetry.

For mechanically-exfoliated layered materials, their naturally irregular geometries inherently lack rotational symmetry. The situation remains with the introduction of a Gaussian light source, which is associated with a rotational symmetric profile and thereby could not provide complementary handedness. On the other hand, for regular-shaped materials possessing axes of symmetry, translational motions free of orientation changes can be produced with a Gaussian-profiled illumination, with the requirement that the light spot center should coincide with one of the symmetry axes (as illustrated in Fig. 3 the main text). However, fulfilling this requirement in experiments becomes challenging due to factors such as limited accuracy of imaging and translation stages, the machining error or slight misalignments in the optical path, which prevent a focused light spot from being perfectly Gaussian. Hence, even with the intention of inducing purely translational motion of motors, in-plane rotation would still occur as a byproduct, which explains its frequent appearance in the OTE actuation experiments.

To theoretically account for the locomotion mode of in-plane rotation, multiphysics simulations are performed on a pedagogical model shown in Fig. S9a. Specifically, the simulation model incorporates a square-shaped gold plate and a Gaussian light spot, which is deviated from both the centroid and axes of symmetry of the gold plate. Upon the delivery of a single light pulse, non-rigid body displacements are generated across the entire plate, which in turn induce friction force that inclines to resist the movements of each individual contact area. The X and Y components of displacements (u and v) as well as the total friction force exerted on the plate are extracted from three probing points (denoted in Fig. S9a) and plotted in Fig. S9b and c. As discussed in the main text, owing to the strong damping effects of friction force on photothermally-excited elastic waves, non-static components of both the elastic displacement and the induced friction force would dissipate rapidly within a brief time interval of a few tens of nanoseconds. In particular, the motion of the centroid, as well as the friction force applied on the gold plate in the direction of motion, are fundamentally linked to the dynamics of non-static elastic waves, and hence should be settled at an early stage before the complete attenuation of said waves. After the dissipation of non-static components, the motion behavior would transition to

a semi-static state with a balanced friction force.

In contrast to the simulation results presented in the main text, when an initial bias is applied to break the rotational symmetry (which also provides the bias to enable translational motion), the gold plate not only experiences center of mass motion, causing its centroid to move towards the light spot center, but also undergoes a slight rotation around the Z axis. The in-plane rotation at the x-y plane is manifested as the misalignment of elastic displacements (along both X and Y axes) among three probing points at the end of the pulse-wise step motion (Fig. S9b and c). The suggested nonuniformity in the elastic displacement across the gold plate, and inevitably in the accompanying friction force induced by the former on the contact surface, can be better visualized in Fig. S9d and e, in which the two quantities exhibit clear vortical profiles corresponding to a clockwise rotation. From the perspective of Newton's law of motion, the friction force, apart from being responsible for the center of mass motion, should also provide the torque which enables in-plane rotation of the gold plate around the Z axis. The torque is integrated on the contact surface and calculated as $Total\ \boldsymbol{M_z} = \int \boldsymbol{M_z} \cdot dS = \int \boldsymbol{r}_{||} \times (\boldsymbol{F}_{fric})_{||} \cdot dS$, where $\boldsymbol{r}_{||} = \boldsymbol{X} + \boldsymbol{Y}$ and $(\boldsymbol{F}_{fric})_{||} = (\boldsymbol{F}_{fric})_x + (\boldsymbol{F}_{fric})_y$, denoting the position vector and the in-plane friction force in each unit area dS on the contact surface, respectively. To decouple the in-plane rotation from the translational motion, while calculating the torque, the centroid is set to be the reference point, and the coordinate in the material frame (as compared to that in the geometry frame) is employed which remain constant despite the displacements. The as-calculated torque as a function of time is plotted in Fig. S10. Similar to the calculated friction force, the torque dissipates rapidly along with the dissipation of elastic waves. Specifically, a torque pulse along the negative Z axis, which corresponds to a clockwise rotation, is generated in the initial few nanoseconds and predominate the remaining pulses, the latter relating to edge reflections of initially-excited elastic waves.

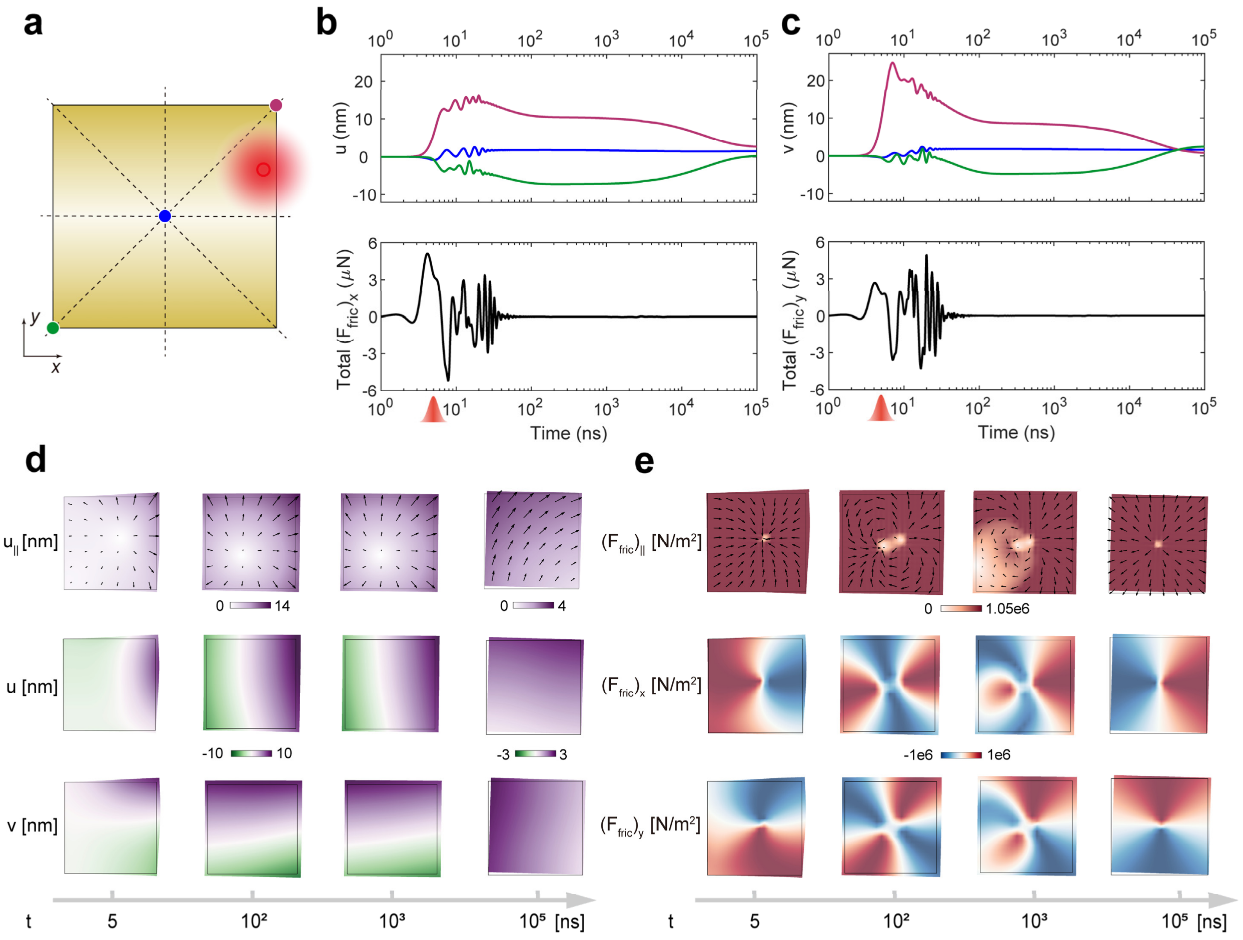

Figure S9. Theoretical calculations incorporating in-plane rotations in a rotational symmetry-breaking configuration. a. Schematic of the simulation model. The light spot is highlighted in red, and the dashed lines denote the axes of symmetry of the square gold plate. **b, c**. Calculation results showing evolutions of the (**b**) x and (**c**) y components of displacement (upper panel) and total friction force (lower panel) probed at three different points with the delivery of a single light pulse. The green, blue and purple curves correspond to the probing positions on the contact surface denoted in **a**. Components of the total friction force are derived through integration of the friction force per unit area (with shear strength of friction = $1e^6\ N/m^2$) on the contact surface. **d, e**. Time-evolving profiles of the (**d**) elastic displacement and (**e**) friction force in the *x-y* plane. The in-plane components $u_{||} = u\hat{x} + v\hat{y}$ and $(F_{fric})_{||} = (F_{fric})_x\hat{x} + (F_{fric})_y\hat{y}$ are displayed in the upper panel, while the *x* (u; $(F_{fric})_x$) and *y* (v; $(F_{fric})_y$) components are displayed in the middle and lower panels, respectively. In the simulation settings, the square plate has 4 μm side length and 100 nm thickness; the $1/e$ diameter of the light spot is 2 μm; the light source has a pulse width equal to 2 ns and the single pulse energy is 25 nJ.

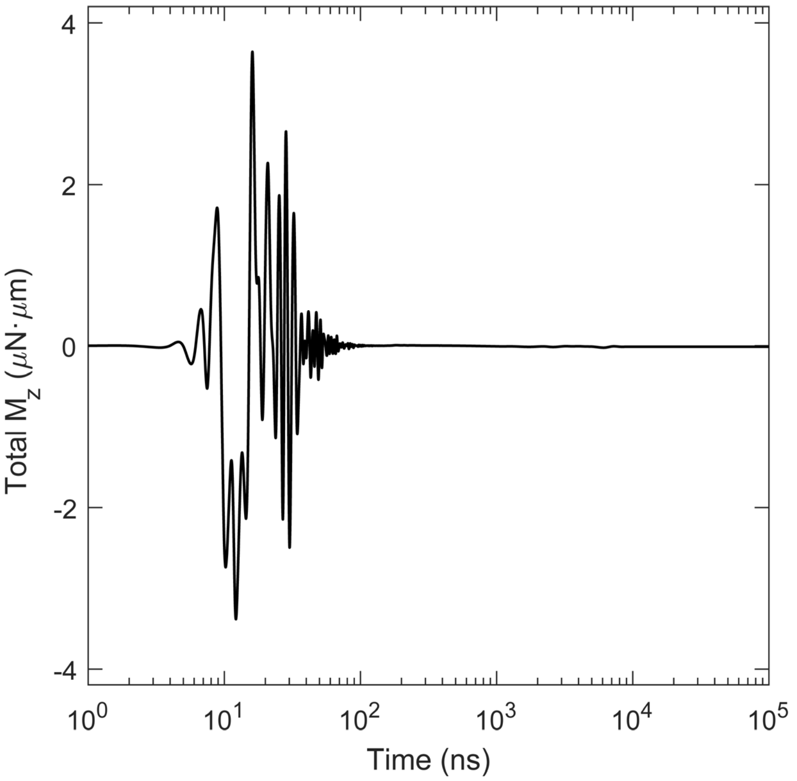

Figure S10. Time-evolution of the out-of-plane torque integrated on the contact surface. To rule out the influence of the center of mass motion, the torque is calculated using the coordinate in the material system rather than in the geometry system. The simulation settings are the same as in Fig. S9.

1 Gusev, V. E. & Karabutov, A. A. Laser optoacoustics. *NASA STI/Recon Technical Report A* **93**, 16842 (1991).

2 Landau, L. D., Lifshitz, E. M., Kosevich, A. d. M. & Pitaevskii, L. P. *Theory of elasticity: volume 7*. Vol. 7 (Elsevier, 1986).

3 Agustín, S. On thermal diffusivity. *European Journal of Physics* **24**, 351, doi:10.1088/0143-0807/24/4/353 (2003).

4 Qiannan, J., Weiwei, T., Wei, Y. & Min, Q. Fibre tapering using plasmonic microheaters and deformation-induced pull. *Light: Advanced Manufacturing* **4**, 25-36 (2023).

5 Lu, J. *et al.* Light-induced reversible expansion of individual gold nanoplates. *AIP Advances* **7**, doi:10.1063/1.4998703 (2017).

Movie S1: OTE actuation of a gold plate on a quartz glass substrate (supplementing Fig. 1e)

To facilitate observation of the OTE motion, the video is adjusted to play at twice the original speed. The translation stage is set to move in a stepwise manner at a precision of 1 μm/step.

Movie S2: OTE actuation of a fragment of exfoliated graphite on a silicon substrate (supplementing Fig. 1f)

To facilitate observation of the OTE motion, the video is adjusted to play at twenty times the original speed. The translation stage is set to move in a stepwise manner at a precision of 100 nm/step.

Movie S3: Synchronized motion of a gold plate and a translation stage at a uniform speed of 11 $\mu m/s$ (supplementing the data point in Fig. 2f at 20 kHz repetition rate)

The video plays at its originally recorded speed. The translation stage is set to move continuously at a fixed velocity of 11 $\mu m/s$.
